# Targeting DDX3 with a small molecule inhibitor for lung cancer therapy

**DOI:** 10.15252/emmm.201404368

**Published:** 2015-03-27

**Authors:** Guus M Bol, Farhad Vesuna, Min Xie, Jing Zeng, Khaled Aziz, Nishant Gandhi, Anne Levine, Ashley Irving, Dorian Korz, Saritha Tantravedi, Marise R Heerma van Voss, Kathleen Gabrielson, Evan A Bordt, Brian M Polster, Leslie Cope, Petra van der Groep, Atul Kondaskar, Michelle A Rudek, Ramachandra S Hosmane, Elsken van der Wall, Paul J van Diest, Phuoc T Tran, Venu Raman

**Affiliations:** 1 Department of Radiology and Radiological Science, Johns Hopkins University School of MedicineBaltimore, MD, USA; 2 Department of Pathology, University Medical Center UtrechtUtrecht, The Netherlands; 3 Department of Radiation Oncology, Johns Hopkins University School of MedicineBaltimore, MD, USA; 4 Department of Molecular and Comparative Pathobiology, Johns Hopkins University School of MedicineBaltimore, MD, USA; 5 Department of Anesthesiology, University of Maryland School of MedicineBaltimore, MD, USA; 6 Department of Oncology, Johns Hopkins University School of MedicineBaltimore, MD, USA; 7 Department of Chemistry & Biochemistry, University of MarylandBaltimore County, MD, USA; 8 Department of Internal Medicine, University Medical Center UtrechtUtrecht, The Netherlands

**Keywords:** DDX3, DNA repair, lung cancer, radiation-sensitizing agent, small molecule inhibitor

## Abstract

Lung cancer is the most common malignancy worldwide and is a focus for developing targeted therapies due to its refractory nature to current treatment. We identified a RNA helicase, DDX3, which is overexpressed in many cancer types including lung cancer and is associated with lower survival in lung cancer patients. We designed a first-in-class small molecule inhibitor, RK-33, which binds to DDX3 and abrogates its activity. Inhibition of DDX3 by RK-33 caused G1 cell cycle arrest, induced apoptosis, and promoted radiation sensitization in DDX3-overexpressing cells. Importantly, RK-33 in combination with radiation induced tumor regression in multiple mouse models of lung cancer. Mechanistically, loss of DDX3 function either by shRNA or by RK-33 impaired Wnt signaling through disruption of the DDX3–β-catenin axis and inhibited non-homologous end joining—the major DNA repair pathway in mammalian somatic cells. Overall, inhibition of DDX3 by RK-33 promotes tumor regression, thus providing a compelling argument to develop DDX3 inhibitors for lung cancer therapy.

## Introduction

Lung cancer is the most common cancer worldwide, and it claims more lives than prostate, colon, and breast cancer combined (Siegel *et al*, 2013). Depending on tumor type and stage, the treatment for lung cancer patients typically consists of surgery or chemoradiation. For most patients, current treatments do not cure the disease and are associated with substantial toxicity. Although surgical resection offers the best long-term survival for lung cancer patients, only a subset of these patients are considered operable and chemoradiation is the only option for the majority of patients (Manser *et al*, 2005).

Recent advances in radiation therapy such as stereotactic body radiation therapy (SBRT) or stereotactic ablative radiation therapy (SABR) have shown increased efficacy to reduce lung tumor burden and offer a new therapeutic modality to non-surgical patients. Clinical experiences with SBRT in early-stage lung cancer and oligometastatic cancer have demonstrated excellent local control of greater than 90% (Timmerman *et al*, 2010). Because of increased toxicity with delivery of SBRT to large treatment targets or following re-treatment, there has been an ongoing search for tumor-selective radiation sensitizers that would enable the use of lower dose per fraction with increased efficacy (Senthi *et al*, 2012).

In our quest to characterize cellular pathways that are essential for the oncogenic state, we have identified DDX3, an RNA helicase, which is dysregulated in many cancer types including lung cancer. DDX3 is a member of the DEAD-box family which is involved in a number of cellular processes like transcription, RNA splicing, mRNA export, and translation initiation (Lorsch, 2002; Rocak & Linder, 2004). DDX3 has also been associated with cancer biogenesis (Hu *et al*, 2004). Previously, we identified DDX3 in a microarray screen of breast cancer cells exposed to cigarette smoke and demonstrated its role in cancer progression (Botlagunta *et al*, 2008). DDX3 promotes proliferation and cellular transformation (Hu *et al*, 2004; Shih *et al*, 2007; Lee *et al*, 2008), has anti-apoptotic properties (Li *et al*, 2006; Sun *et al*, 2008, 2011), modulates cell adhesion and motility (Chen *et al*, 2014), and responds to hypoxia via HIF-1α (Botlagunta *et al*, 2011; Bol *et al*, 2013). Besides the oncogenic role of DDX3 in cancer biogenesis, there is a report that indicates loss of DDX3 via p53 inactivation can promote tumor malignancy in non-small cell lung cancer (Wu *et al*, 2014).

Also, recent evidence has identified that DDX3 acts as an allosteric activator of casein kinase 1 in the Wnt/β-catenin pathway (Cruciat *et al*, 2013). Initially, the Wnt/β-catenin pathway was described in colon cancer. Activating mutations of DDX3 were also shown to be involved in pathogenic Wnt pathway activation in medulloblastoma (Jones *et al*, 2012; Pugh *et al*, 2012; Robinson *et al*, 2012) and chronic lymphatic leukemia (CLL) (Wang *et al*, 2011). Recently, it has been shown that activated Wnt signaling predicts decreased survival in lung cancer patients (Xu *et al*, 2011; Shapiro *et al*, 2013) and decreases sensitivity to radiation therapy (Woodward *et al*, 2007; Zhang *et al*, 2010).

In the present study, we synthesized a DDX3 inhibitor, RK-33 (diimidazo[4,5-*d*:4′,5′-*f*]-[1,3]diazepine) (Kondaskar *et al*, 2010) which can potentially be used in cancer treatment. Binding of RK-33 to DDX3 impedes the function of DDX3, resulting in activation of cell death pathways, inhibition of the Wnt-signaling pathway, and abrogation of non-homologous end-joining (NHEJ) activity. In combination with radiation, synergistic cell death effects were observed both *in vitro* and in multiple preclinical lung cancer models.

## Results

### DDX3 overexpression correlates with aggressive lung cancer

**Figure 1:**
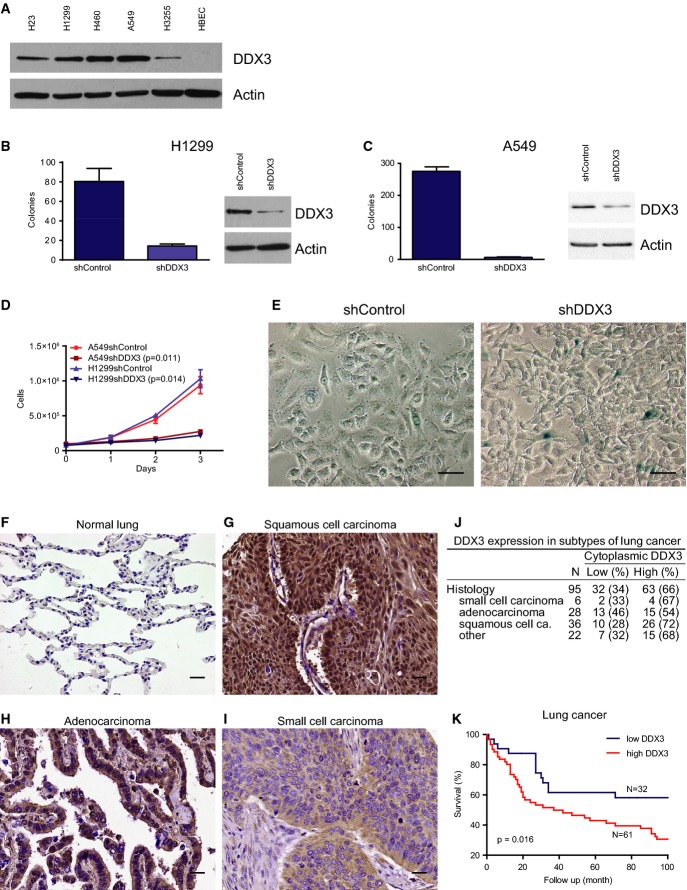
DDX3 expression and knockdown phenotype in lung cancer cell lines and in lung cancer patient samples

DDX3 is expressed in lung cancer cell lines (H23, H1299, H460, A549, and H3255) but not in the normal lung cell line HBEC (Fig1A). To assess the effect of DDX3 on malignant growth, we generated two cell lines with reduced DDX3 expression—H1299shDDX3 and A549shDDX3. Parental H1299 and A549 cells, transfected with vector control, efficiently form colonies and grow rapidly. However, knockdown of DDX3 significantly reduced colony formation (Fig1B and C) and proliferation (Fig1D) and resulted in a higher percentage of cells undergoing senescence (Fig1E).

A Immunoblot of DDX3 expression in lung cancer cell lines.

B, C Colony-forming assays in H1299 (B) and A549 (C) lung cancer cells after knockdown by shRNA lentiviral constructs designed against DDX3 or vector control. Corresponding immunoblots displaying knockdown levels of DDX3. Mean from 3 replicates with SD.

D Proliferation of A549 and H1299 cells after knockdown of DDX3. Mean from 3 replicates with SD. (A549 *P *= 0.011, H1299 *P *= 0.014; exponential curve fit, extra sum of squares *F*-test).

E β-galactosidase staining in parental A549 cells and A549 DDX3 knockdown cells displaying senescent cells identified by the blue color.

F Expression of DDX3 by immunohistochemistry in normal lung tissue.

G DDX3 expression in squamous cell carcinoma.

H DDX3 expression in adenocarcinoma.

I DDX3 expression in small cell carcinoma.

J Expression of DDX3 in different histological types of lung cancer. All data sets were compared against each other (chi-square test, *P *= 0.481).

K Survival analysis of lung cancer patients in low and high DDX3 expressing tumors (Kaplan–Meier curve and log-rank test, *P *= 0.016). Data information: Scale bars: 25 μm. Source data are available online for this figure.

To corroborate our findings in lung cancer patients, we analyzed 95 lung cancer samples for DDX3 expression. In normal lung parenchyma, we saw little or no expression of cytoplasmic DDX3 (herein DDX3 expression) (Fig1F). However, almost all (94 out of 95) lung cancer samples expressed DDX3, of which 63 samples (66%) expressed high levels of DDX3 (Fig1G–J). High DDX3 expression was equally distributed among different histological subtypes of lung cancer including NSCLC and SCLC (Fig1J). Patients whose lung cancer samples expressed high levels of DDX3 died on an average 18 months earlier as compared to patients with low DDX3-expressing tumors (Fig1K). The hazard ratio (HR) for death was 2.10 (95% CI; 1.13–3.93).

Furthermore, DDX3 was found to be a predictor of overall survival, independent of tumor size, grade, and histological type by multivariable analysis (Table1A and B). In addition, analysis of gene signatures in human cancers indicates that high DDX3 expression correlates with shorter overall survival in NSCLC (Supplementary Fig S1) (Bild *et al*, 2006). These results indicate that DDX3 is essential for cancer cell proliferation and survival, especially in aggressive subtypes of lung cancer, and may be an important molecular determinant of lung cancer survival.

**Table 1:** Comparison of DDX3 expression with clinical parameters and survival analysis

A						
Average	Cytoplasmic DDX3			Nuclear DDX3		
	Low (0–1)	High (2–3)	*P*-value	< 10%	≥ 10%	*P*-value
*N*	32	63		90	5	
Mean age (range)	61.6 (36–78)	63.3 (38–79)	0.443^#^	62.4 (36–79)	67.8 (58–79)	0.249^#^
Mean tumor size (range)	4.2 (1.3–10.0)	3.8 (0.9–10.0)	0.380^#^	4.0 (0.9–10.0)	3.5 (1.6–4.7)	0.612^#^
Histological type						
Small cell carcinoma	33.3% (2)	66.7% (4)	0.481	100% (6)	0% (0)	0.075
Squamous cell carcinoma	27.8% (10)	72.2% (26)		86.1% (31)	13.9% (5)	
Adenocarcinoma	46.4% (13)	53.6% (15)		100% (28)	0% (0)	
Other	31.8% (7)	68.2% (15)		100% (22)	0% (0)	
Grade						
1	0% (0)	100% (2)	0.758	100% (2)	0% (0)	0.560
2	37.5% (12)	62.5% (20)		90.6% (29)	9.4% (3)	
3	37.5% (12)	62.5% (20)		96.9% (31)	3.1% (1)	
4	25% (3)	75% (9)		100% (12)	0% (0)	
Gender						
Male	35.1% (27)	64.9% (50)	0.770	93.5% (72)	6.5% (5)	0.583^$^
Female	31.2% (5)	68.8% (11)		100% (16)	0% (0)	
Stage (pathologic)						
I & II	38.9% (21)	61.1% (33)	0.403	92.6% (50)	7.4% (4)	0.571^$^
III & IV	28.6% (6)	71.4% (15)		100% (21)	0% (0)	

B				
Variables	Univariate		Multivariate	
	HR (95% CI)	*P*-value	HR (95% CI)	*P*-value
Tumor size	1.078 (0.940–1.236)	0.283		
Histological type	1.579 (0.846–2.950)	0.152	1.488 (0.759–2.916)	0.247
Grade	1.418 (0.954–2.108)	0.084	1.367 (0.939–1.991)	0.102
Cytoplasmic DDX3	2.103 (1.126–3.930)	0.020	2.136 (1.078–4.233)	0.030

(A) Baseline characteristics of differentially expressed cytoplasmic and nuclear DDX3 in lung cancer patient samples (*P*-values are determined by chi-square test unless otherwise indicated: # = *t*-test; $ = Fisher's exact test). (B) Univariate and multivariate cox regression analyses in lung cancer patient samples on clinically relevant variables related to aggressiveness (tumor size, histological type, and grade). Tumor size was not used in multivariate regression analysis as it has a univariate *P*-value > 0.2 and made the multivariate regression model less predictive.

### RK-33 binds to DDX3 and decreases its helicase function

**Figure 2:**
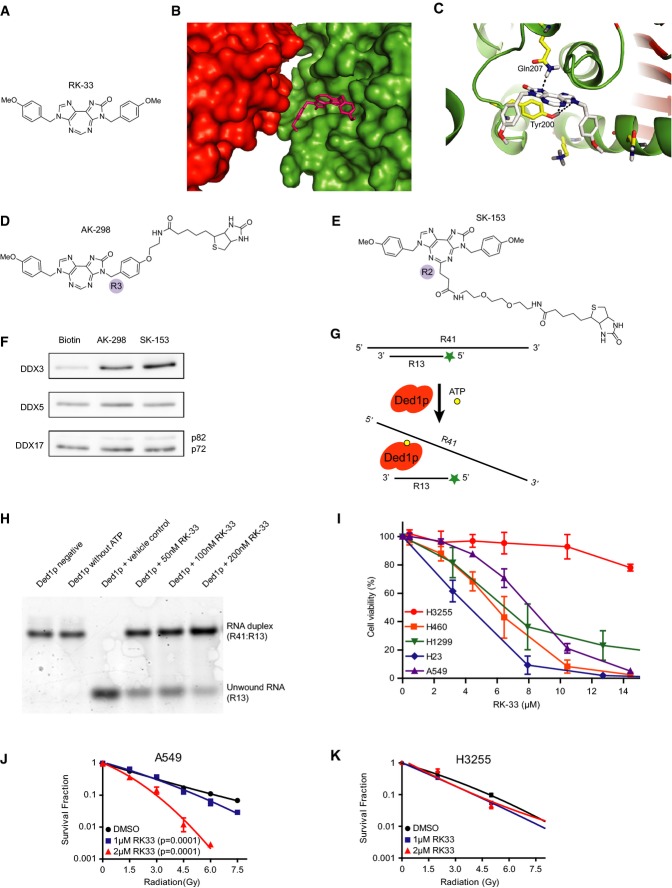
Specific binding of RK-33 to DDX3 and induction of radiosensitization in lung cancer cell lines

Based on the role of DDX3 in proliferation and as a potential marker of aggressive cancer, we rationally designed small molecules to bind specifically to the ATP-binding cleft of DDX3 (Kondaskar *et al*, 2010). We identified a fused diimidazodiazepine molecule (RK-33; Fig2A) that exhibited promising cell death kinetics and has a computed binding affinity of −8 kcal/mol between RK-33 and DDX3 (Fig2B and C). To evaluate binding of RK-33 to DDX3, we synthesized two biotinylated RK-33 molecules (Fig2D and E) and demonstrated that RK-33 binds specifically to DDX3, but not to the closely related proteins DDX5 and DDX17 (Fig2F).

A Chemical structure of RK-33.

B A predicted molecular model of RK-33 docked into the ATP-binding cleft of DDX3. RK-33 is displayed in pink, the surface of the DEADc domain is in green, and the surface of the HELICc domain is in red.

C Hydrogen bond interactions between RK-33 and DDX3. Alpha helices are displayed in green, and β-sheets are shown in maroon.

D, E Chemical structures of biotin-linked RK-33 at R3 position with ethylene amine linker (AK-298) and biotin-labeled RK-33 at R2 position with (PEG)2 ethylene amide linker (SK-153). The two structural differences of AK-298 and SK-153 are the length of the biotin linker and attachment position at RK-33.

F Immunoblots of pull-down assay of DDX3 with biotin, AK-298, and SK-153. Lower panels display results using DDX5 and DDX17 antibodies.

G Schematic representation of helicase assay.

H Immunoblot displaying increasing concentrations of RK-33 (50, 100, 200 nM) resulting in increased inhibition of unwinding of oligomer products (lanes 4–6).

I MTS viability assay of various lung cancer cell lines treated with RK-33 for 72 h. Mean from 3 replicates with SD.

J, K Colony-forming assay of A549 and H3255 cells treated with RK-33 and with various doses of radiation 4 h later. Curves were fitted with a quadratic polynomial equation. Mean from 2 replicates with SD. *P*-values were determined by the extra sum of squares *F*-test. Source data are available online for this figure.

To establish whether RK-33 can perturb the helicase activity of DDX3, we carried out helicase assays as described (Sengoku *et al*, 2006, Fig2G and H). RK-33 significantly reduced the unwinding activity of Ded1p (yeast homolog of DDX3), in a dose-dependent manner, starting with as little as 50 nM.

### RK-33 inhibits cancer growth and radiosensitizes lung cancer cells in a DDX3-dependent manner

To evaluate whether inhibition of DDX3 by RK-33 would lead to cancer cell cytotoxicity, we assessed cell viability in various lung cancer cell lines (Fig2I). Cancer cell lines with high levels of DDX3 expression (A549, H1299, H23, and H460) were more sensitive to RK-33 (IC_50_ = 4.4–8.4 μM) as compared to H3255, a cell line with low DDX3 expression (IC_50_ > 25 μM). Percentage of cells undergoing early apoptosis (Annexin V positive) and late apoptosis (PI positive) is shown in Supplementary Fig S2.

Since radiation therapy is one of the mainstays for treatment of lung cancer, we assessed the combination effect of RK-33 and radiation. We carried out colony-forming assays to establish the response of A549 cells (high DDX3 expression) and H3255 cells (low DDX3 expression) to radiation with or without RK-33 (Fig2J and K). Not only did RK-33 cause cytotoxicity in A549 cells, but at low concentrations of 1 μM RK-33 (*P* = 0.0001) and 2 μM RK-33 (*P* = 0.0001), it also sensitized A549 cells to γ-radiation. H3255 cells (low DDX3 expression), on the other hand, were not sensitized to γ-radiation by RK-33 (1 μM, *P* = 0.095; 2 μM, *P* = 0.065).

### DDX3 knockdown and RK-33 perturb common gene regulatory pathways

**Figure 3:**
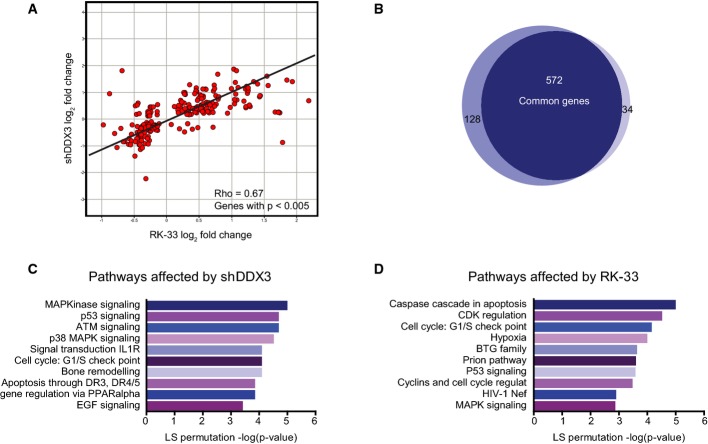
Bioinformatics analysis of DDX3 knockdown and RK-33 treatment

To confirm the inhibition of DDX3 by RK-33 and determine specificity, we measured gene expression in MDA-MB-231 cells by microarray analysis after treatment of RK-33 or knockdown of DDX3. As shown in Fig3A, gene expression changes of RK-33-treated cells correlated with DDX3 knockdown cells (rho = 0.673, using genes altered in both classes at *P* < 0.005). This is supported by a Venn diagram displaying the overlap of gene expression between shDDX3- and RK-33-treated cells (Fig3B). This indicates that the functional activity of RK-33 is via DDX3 inhibition and that it could be used as a small molecule inhibitor of DDX3.

A Scatter plot of the gene expression log2 fold change in DDX3 knockdown and RK-33-treated MDA-MB-231 cells. Each red dot represents a gene, which was significantly perturbed after treatment with RK-33 and after knockdown of DDX3.

B The Venn diagram depicts the number of common genes dysregulated by both shDDX3 and RK-33 treatments.

C, D BioCarta pathway analysis of gene expression in DDX3 knockdown and RK-33-treated cells. Pathways are ranked on LS permutation *P*-values from top to bottom.

To further elucidate the cytotoxic mechanism of RK-33, we explored gene expression patterns by microarray analysis of DDX3 knockdown cells and cells treated with RK-33. We found that the mechanism behind the decreased cellular proliferation perhaps could be assigned to reduced cell cycle progression and inhibition of the MAPK pathway (Fig3C and D).

**Figure 4:**
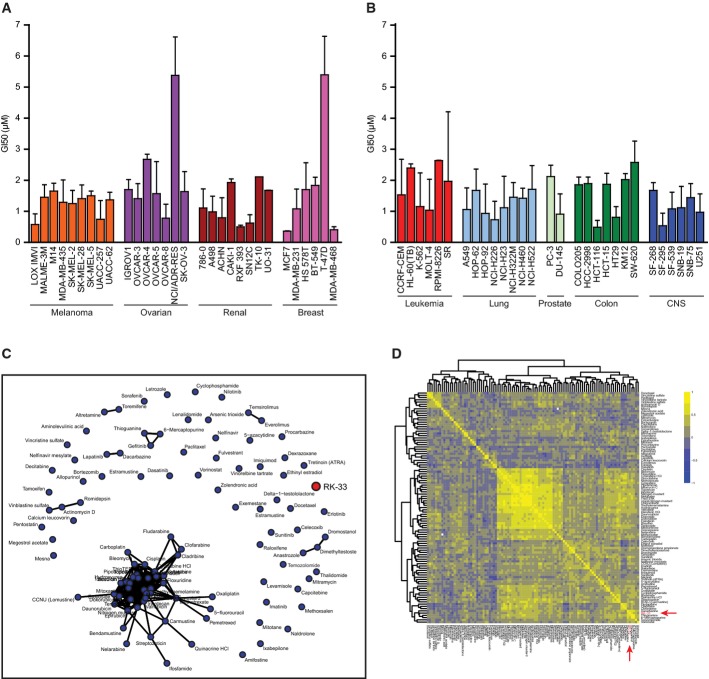
Comparison of the GI_50_ values of RK-33 with FDA-approved drugs on the NCI-60 panel of cell lines

To assess the effect of RK-33 on a wide variety of cell lines, we tested the NCI-60 panel of cell lines (Shoemaker *et al*, 1988; Shoemaker, 2006) for a decrease in cellular growth (Fig4A and B). Next, we compared the growth inhibition of the NCI-60 cell lines by RK-33 with that of 102 common FDA-approved drugs using network analysis (Fig4C). A well-connected sub-network in the middle of the plot indicates that all of these drugs have similar patterns of sensitivity across the cell lines. RK-33 and several other agents are not connected to networks, indicating that none of these have near-neighbors among FDA-approved drugs in cancer. We also performed an unsupervised cluster analysis of the 102 FDA-approved drugs based on the correlation structure of the GI_50_ levels (Fig4D). RK-33 sits in the bottom right corner in a small cluster of weak-to-moderately correlated agents including dacarbazine, thioguanine, temozolomide, and vorinostat, supporting the distinctive working mechanism of RK-33 as compared to other drugs.

A, B The graph depicts the growth inhibitory properties (GI50) of RK-33 for the NCI-60 panel of cell lines. The NCI-60 is a panel of 60 extensively characterized human cell lines derived from nine distinct tumor types: melanoma, ovarian, renal, breast, leukemia, lung, prostate, colon, and CNS.

C Network analysis of 102 FDA-approved drugs and RK-33 based on GI_50_ in the NCI-60 cell line panel.

D Unsupervised cluster analysis of the 102 FDA-approved drugs based on the correlation structure of the GI_50_ levels. The result is shown as a symmetric heat map with positive associations depicted in yellow and negative associations shown in blue. Data information: Error bars represent SD and all experiments were done in replicates.

### Toxicology, biodistribution, pharmacokinetics, and metabolism of RK-33

**Figure 5:**
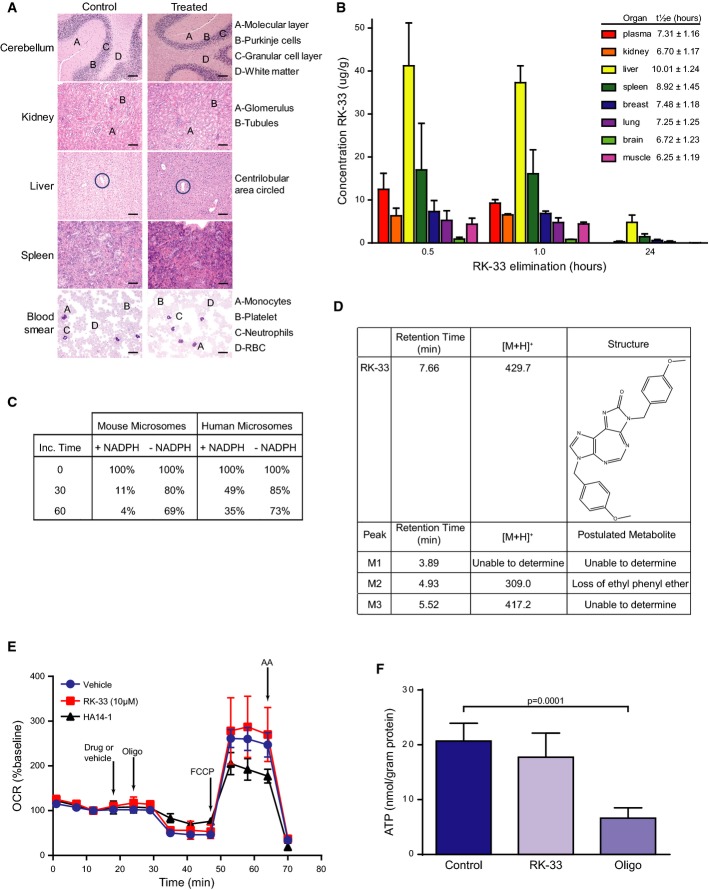
Toxicity studies of RK-33 in mice

Prior to initiating the animal experiments, we carried out toxicology, biodistribution, and metabolism assessments of RK-33. Toxicology studies indicated that RK-33, at the dose used, was non-toxic in SCID mice. As shown in Fig5A, histopathology of the different tissues from control (DMSO) and RK-33-treated mice did not exhibit any discernable morphological changes. Biodistribution studies revealed that RK-33 was able to accumulate at therapeutic dose in various organs, thus enhancing the clinical relevance for the use of RK-33 as chemotherapeutic agent (Fig5B). Moreover, NADPH-independent and NADPH-dependent metabolism was observed when RK-33 was incubated with human liver and mouse microsomes (Fig5C). Subsequent chromatography analysis identified three potential metabolites (Fig5D). In addition, liver and kidney function tests, as well as the blood and lipid profiles, were not altered between the control and RK-33-treated groups (Table2).

**Table 2:** Blood toxicity studies

Blood samples of RK-33-treated SCID mice			
	DMSO ± SD	RK-33 ± SD	Normal range
Blood cells			
RBC (M/μl)	10.48 ± 0.55	10.01 ± 0.77	6.36–9.42
Hb (g/dl)	15.2 ± 0.8	14.7 ± 0.6	11.0–15.1
MCV (fl)	52.2 ± 0.9	55.1 ± 1.3	45.4–60.3
WBC (K/μl)	4.14 ± 1.12	5.83 ± 2.61	1.8–10.7
Thrombocytes (K/μl)	1267 ± 196	746 ± 185	592–2,972
Liver biochemical values			
ALT (U/l)	46 ± 8	72 ± 34	20–80
AST (U/l)	81 ± 3	224 ± 152	50–300
ALP (U/l)	82 ± 4	69 ± 5	28–96
Kidney biochemical values			
BUN (mg/dl)	18 ± 1.0	22 ± 2.0	17–31
Creatinine (mg/dl)	0.30 ± 0.00	0.35 ± 0.05	0.3–1.0
Calcium (mg/dl)	9.2 ± 0.1	9.1 ± 0.2	9.0–13.0
Albumin (g/dl)	3.05 ± 0.25	3.05 ± 0.05	2.5–4.8
Lipids and other biochemical values			
Cholesterol (mg/dl)	123 ± 10	120 ± 1	60–165
Triglycerides (mg/dl)	141 ± 38	215 ± 14	109–172
Amylase (U/l)	918 ± 13	905 ± 42	1,063–1,400
Glucose (mg/dl)	151 ± 5	166 ± 4	62–175

The table displays blood, liver, kidney, and lipid toxicity data from RK-33-treated SCID mice. RBC, red blood cells; Hb, hemoglobin; MCV, mean corpuscular volume; WBC, white blood cells; ALT, alanine aminotransferase; AST, aspartate transaminase; ALP, alkaline phosphatase; BUN, blood urea nitrogen.

Following injection of 20 mg/kg of RK-33, twice a week for 7 weeks, extensive histopathological examination was carried out following necropsy. Identical patterns were observed both in the control and in the treated mice (*n* = 2). Samples were stained with H&E. Scale bar is 50 μm.

Pharmacokinetics of RK-33 in SCID mice at various time intervals. Results are mean ± SD from 5 mice. LC-MS/MS method was used to determine concentration of RK-33 in mouse plasma and tissue.

Liquid chromatography–mass spectrometry (LC-MS/MS) analysis was performed to determine different metabolites of RK-33.

RK-33 and metabolites characterized by LC-MS/MS in human liver microsomes using the scan mode function of the LC-MS/MS.

HAPI cells were treated with RK-33 (10 μM), HA14-1 (25 μM), or DMSO vehicle, followed by oligomycin (oligo, 0.5 μg/ml), FCCP (3 μM), and antimycin A (AA, 1 μM) while oxygen consumption rate (OCR) was measured. Pyruvate (10 mM) was added in combination with FCCP to ensure that substrate supply was not rate-limiting for maximal OCR. Data are mean ± SD from 2 to 3 wells and representative of independent experiments performed with two different HAPI passages. OCR is baseline-normalized to the point prior to drug or vehicle addition.

HAPI microglial cells were incubated for 1 h in glucose-free XF24 assay medium that was supplemented with 2-deoxyglucose (50 mM) and pyruvate (10 mM). RK-33 (10 μM), oligomycin (0.5 μg/ml), or vehicle control was additionally present as indicated. Results are mean ± SD from 12 replicates pooled from experiments using two consecutive passages. Significance was assessed by two-sided, unpaired *t*-test.

### RK-33 does not perturb mitochondrial functions

To test whether therapeutically relevant concentrations of RK-33 interfere with mitochondrial function, RK-33 (5 or 10 μM) or vehicle was added to the highly aggressive proliferating immortalized (HAPI) cell line (Cheepsunthorn *et al*, 2001), while cellular oxygen consumption was measured. The ATP synthase inhibitor oligomycin (oligo), the uncoupler FCCP, and the electron transport chain inhibitor antimycin A (AA) were added subsequent to RK-33. Drugs that uncouple oxidative phosphorylation from electron transport increase ATP synthesis-independent oxygen consumption measured in the presence of oligomycin. Drugs that inhibit electron transport impair maximal respiration measured in the presence of the uncoupler FCCP. RK-33 at 5 (not shown) or 10 μM (Fig5E) failed to alter baseline oxygen consumption rate (OCR), OCR measured in the presence of oligomycin (oligo), or OCR measured in the presence of the uncoupler FCCP. As a positive control, HA14-1 (25 μM), a Bcl-2 inhibitor, which is known to cause both mitochondrial uncoupling and respiratory inhibition (Milanesi *et al*, 2006), elevated oligomycin-insensitive oxygen consumption and decreased OCR measured in the presence of FCCP. Oxygen consumption in the presence of all drugs was potently inhibited by antimycin A, indicating that it was primarily mitochondrial in origin. To additionally demonstrate that a therapeutically relevant RK-33 concentration does not interfere with mitochondrial function, we evaluated ATP levels in HAPI cells incubated with the cell-permeable mitochondrial complex I substrate pyruvate (10 mM) in the absence of glucose and the presence of the glycolysis inhibitor 2-deoxyglucose (50 mM). Under these conditions, the majority of cellular ATP was generated by mitochondria, as demonstrated by addition of the ATP synthase inhibitor oligomycin, which significantly decreased total cellular ATP by approximately 70%. In contrast to oligomycin, RK-33 (10 μM) failed to significantly decrease ATP. Thus, RK-33 has no effect on either mitochondrial respiration or ATP generation.

### RK-33 in combination with radiation promotes tumor regression in preclinical models of DDX3-overexpressing lung cancer

**Figure 6:**
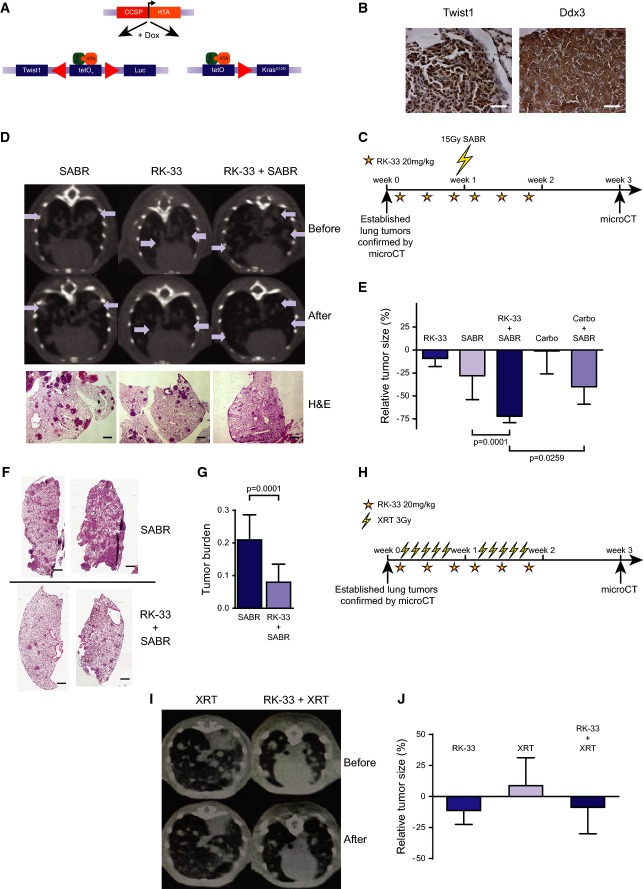
RK-33 induces radiosensitization in preclinical mouse models of lung cancer

To assess whether RK-33 could be a clinically useful radiosensitizer, we evaluated RK-33 in combination with different radiation doses in both an immune competent Twist1/Kras^G12D^ autochthonous lung tumor model (Fig6A) (Tran *et al*, 2012) and an orthotopic human xenograft model for lung cancer. This autochthonous model harbors a Kras^G12D^ mutation and overexpresses Twist1 and Ddx3, which made it a suitable model to test efficacy of RK-33 (Fig6B).

Schematic describing the Twist1/KrasG12D-inducible mouse model.

Confirmation of high expression of Twist1 and DDX3 in the lung tumors of the transgenic Twist1/Kras^G12D^ mouse. Scale bar is 100 μm.

Treatment schedule for mice receiving hypofractionated radiation (SABR). Stars are intraperitoneal (i.p.) injections with RK-33.

Micro-CT images of transgenic Twist1/Kras^G12D^ mice treated as in (C), before treatment and 1 week after treatment. Tumors are indicated by arrows and confirmed by H&E staining of lung sections (lower panel). Scale bar is 250 μm.

Quantification of tumor volume measured by micro-CT in Twist1/Kras^G12D^ mice, as shown in (D). Significance was assessed by two-sided, unpaired *t*-test. Error bars represent SD.

An orthotopic lung tumor model was generated using A549 human lung cancer cells and treated as in (C). Figure displays H&E staining of lung sections from radiation-treated (upper panel) and RK-33- and radiation-treated mice (lower panel). Scale bar is 2 mm.

Quantification of tumor burden (as tumor surface divided by total lung surface) in orthotopic A549 lung cancer mouse model, as shown in (F). Significance was assessed by two-sided, unpaired *t*-test. Error bars represent SD.

Treatment schedule for mice receiving fractionated radiation in 10 fractions. Stars indicate i.p. injections with RK-33. Downward lightning bolts indicate 3-Gy radiation fractions.

Micro-CT images of transgenic Twist1/Kras^G12D^ mice treated as in (H), before treatment and 1 week after treatment.

Quantification of tumor volume measured by micro-CT in Twist1/Kras^G12D^ mice, as shown in (I) and expressed as relative tumor size. Significance was assessed by two-sided, unpaired *t*-test. Error bars represent SEM.

Following tumor formation, the mice were treated either individually or with a combination of RK-33, carboplatin, and radiation as depicted in Fig6C. Tumor progression was followed by micro-computed tomography (micro-CT) imaging before treatment and 1 week after treatment, after which tumor volume was measured and confirmed by H&E staining of lung sections (Fig6D). In addition, we carried out immunohistochemistry analysis for Ki67 expression in these tumors (Supplementary Fig S3). Similar to our *in vitro* results, RK-33 enhanced the radiation effect by 3.7-fold (*P* = 0.0001), which was 1.5-fold more than the radiation sensitization caused by carboplatin (*P* = 0.0259) (Fig6E).

To validate the radiation sensitization by RK-33, we generated an orthotopic lung cancer model by injecting A549 cells into the tail vein of athymic NCr-nu/nu mice. The treatment schedule was similar to that described in Fig6C. Overall, RK-33 significantly enhanced radiation-induced tumor regression (2.6-fold; *P* = 0.0001) in the orthotopic human lung cancer model (Fig6F and G).

Next, we evaluated the effect of RK-33 in our Twist1/Kras^G12D^ lung cancer model with a fractionated treatment regimen (Fig6H). During the 3 weeks following treatment, we saw a modest decrease in tumor growth with radiation and even more so with the combination of RK-33 and radiation; this was however not significant (Fig6I and J). Overall, this demonstrates that RK-33 in combination with hypofractionated radiation, such as used with stereotactic ablative radiation (SABR), effectively decreases lung tumor load in two distinct preclinical lung cancer models and performs significantly better than the commonly used radiosensitizer carboplatin.

### RK-33 induces G1 arrest and causes apoptosis

**Figure 7:**
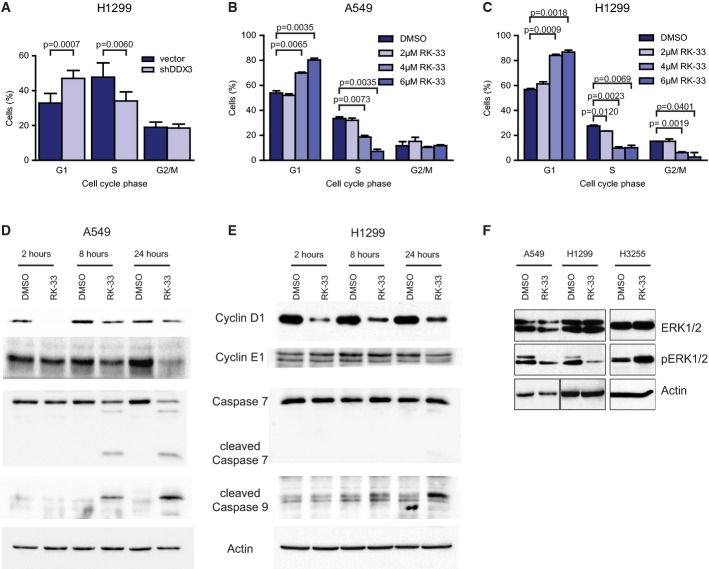
Effect of DDX3 knockdown and RK-33 on cell cycle progression and apoptosis

As loss of DDX3 altered proliferation, we performed cell cycle analysis by flow cytometry, following biological knockdown of DDX3 by shDDX3 and by RK-33 treatment. We found a 13.7% (*P* = 0.006) decrease in S-phase and a 14.1% (*P* = 0.0007) increase in G1-phase in H1299 DDX3 knockdown cells (Fig7A), consistent with a G1 arrest. Similarly, treatment with RK-33 also resulted in a G1 arrest of A549 and H1299 cells in a dose-dependent manner (Fig7B and C).

A Cell cycle analysis of H1299 cells treated with shDDX3 and processed by flow cytometry. Knockdown of DDX3 led to a decrease of cells in S-phase and an increase of cells in G1-phase, indicative of a G1 arrest. Significance was assessed by two-sided, unpaired *t*-test. Error bars represent SD.

B, C Cell cycle analysis of A549 and H1299 cells by flow cytometry after treatment with RK-33 (0, 2, 4, and 6 μM). RK-33 induced a G1 cell cycle arrest in both cell lines. Significance was assessed by two-sided, unpaired *t*-test. Error bars represent SD.

D, E Immunoblot of cell cycle-related proteins (Cyclin D1 and Cyclin E1) and cell death-related proteins (cleaved caspase 7, cleaved caspase 9) in A549 and H1299 cells after treatment with RK-33 (10 μM). Initially, a strong decrease of Cyclin D1 was observed. After 8 and 24 h, cleaved caspases 9 and 7 were apparent.

F Immunoblot of MAPK pathway-related proteins ERK1/2 and phosphorylated ERK1/2 in A549, H1299, and H3255 (RK-33 resistant) cells 24 h after treatment with RK-33 (7.5 μM or 10 μM). ERK2 and especially ERK1 become dephosphorylated after treatment with RK-33 in A549 and H1299 cells but not in H3255 cells. Outlined boxes indicate spliced lanes. Source data are available online for this figure.

Subsequently, we assessed proteins involved in G1/S cell cycle transition (cyclin D1 and E1), apoptosis, and MAPK pathway by immunoblotting. We found a substantial reduction of cyclin D1, which was especially evident 2 h after treatment with RK-33 in A549 and H1299 cells (Fig7D and E). Also, we found cleavage of caspases 7 and 9 in A549 cells (Fig7D) and of caspase 9 in H1299 cells (Fig7E). Moreover, we found a reduction of phosphorylated ERK2 and ERK1 in A549 and H1299 lung cancer cells (high DDX3 expression) and an increase of ERK1/2 phosphorylation in H3255 cells (low DDX3 expression) (Fig7F). Collectively, these results suggest that RK-33 curbs proliferation and induces apoptosis in a DDX3-dependent fashion.

### Wnt signaling is mediated by DDX3 and inhibited by RK-33

**Figure 8:**
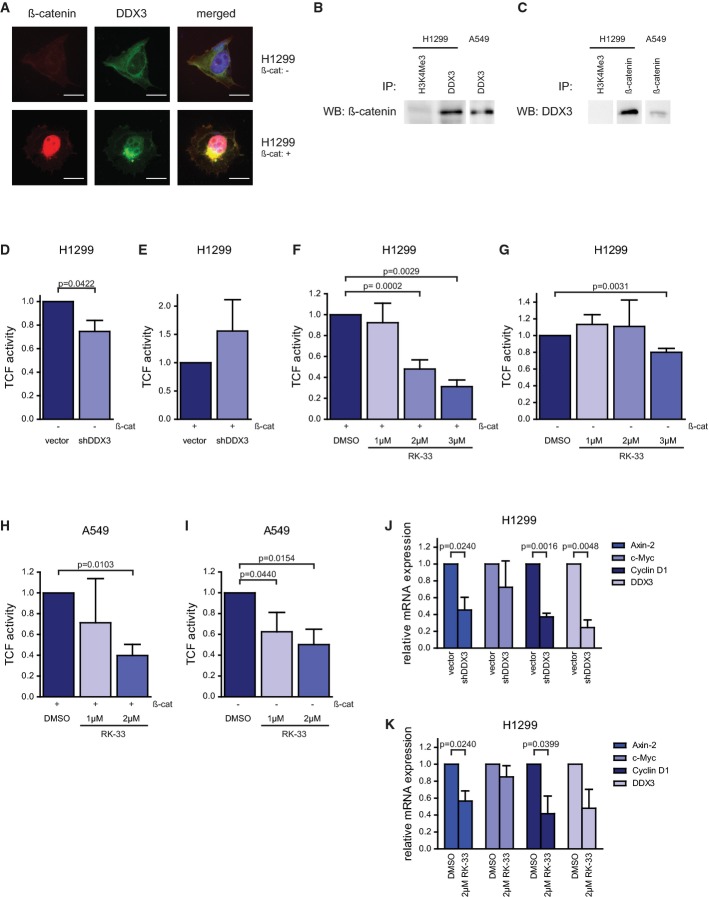
Effect of RK-33 on Wnt signaling via DDX3

As DDX3 is implicated in Wnt signaling (Cruciat *et al*, 2013) and Wnt signaling is an important regulator of proliferation and can cause radiation resistance (Woodward *et al*, 2007; Zhang *et al*, 2010), we evaluated the relation between DDX3/RK-33 and the Wnt pathway. To determine the spatial pattern of DDX3 and β-catenin expression, we carried out immunofluorescence staining for DDX3 and β-catenin in wild-type H1299 cells, as well as in β-catenin-overexpressing H1299 cells. In wild-type H1299 cells, we could detect DDX3 expression in the cytoplasm, but very little nuclear β-catenin staining (Fig8A, top panel). However, forced expression of β-catenin resulted in a relocalization of DDX3 from the cytoplasm to the nucleus (Fig8A, bottom panel). This indicates that DDX3 may act as a transporter protein to shuttle β-catenin in and out of the nucleus. To establish whether there is a physical interaction between DDX3 and β-catenin, we carried out co-immunoprecipitation with DDX3 and β-catenin. As shown in Fig8B and C, DDX3 binds to β-catenin but not to H3K4Me3 (control), which was confirmed by immunoprecipitation using antibodies against both DDX3 and β-catenin.

A β-catenin (red) and DDX3 (green) expression in H1299 cells. After overexpressing β-catenin, both DDX3 and β-catenin accumulate in the nucleus. Scale bar is 10 μm.

B Immunoprecipitation with DDX3 or H3K4Me3 (control) and immunoblotted with β-catenin in A549 and H1299 cells. Outlined boxes indicate spliced lanes.

C Immunoprecipitation with β-catenin or H3K4Me3 (control) and immunoblotted with DDX3 in A549 and H1299 cells. Outlined boxes indicate spliced lanes.

D, E β-catenin/TCF4 activity was determined by the TOP/FOP reporter assay. Co-transfection with β-catenin is indicated below.

F–I H1299 and A549 cells were treated with RK-33 (0, 1, 2, and 3 μM) and co-transfected with β-catenin in (F, H). Treatment with RK-33 decreased TCF4 activity in both cell lines.

J, K Normalized mRNA expression of TCF4-regulated proteins (Axin-2, c-Myc, Cyclin D1) and DDX3 were measured by qRT–PCR in H1299 cells after knockdown of DDX3 (J) and treatment with RK-33 (K). All experiments were repeated three times. Data information: Significance was assessed by two-sided, paired *t*-test. Error bars represent SD. Source data are available online for this figure.

Based on the interaction between DDX3 and β-catenin, we quantified Wnt activity using a TCF reporter assay by transfecting H1299 and H1299 DDX3 knockdown cells with TOP-FLASH or FOP-FLASH constructs (van de Wetering *et al*, 2002). Knockdown of DDX3 decreased TCF activity by 25% (*P* = 0.042) (Fig8D). However, in H1299 cells co-transfected with β-catenin, an increase in TCF activity was observed, but no significant difference was observed by knockdown of DDX3 (Fig8E). RK-33 reduced TCF activity significantly in H1299 and A549 cells, both in wild-type and in β-catenin-overexpressing cells (Fig8F–I).

To confirm the results of the TCF reporter assay, we quantified transcript expression of common TCF-regulated genes—Axin-2, c-Myc, and Cyclin D1—following knockdown of DDX3 as well as treatment with RK-33. As seen in Fig8J, stable knockdown of DDX3 in H1299 cells reduced the expression of Axin-2 (2.2-fold lower; *P* = 0.024), Cyclin D1 (2.7-fold lower; *P* = 0.002), and c-Myc (1.4-fold lower; non-significant). Likewise DDX3 knockdown, RK-33 treatment of H1299 cells reduced the expression of Axin-2 (1.8-fold lower; *P* = 0.024) and Cyclin D1 (2.4-fold lower; *P* = 0.040) (Fig8K).

### Radiation-induced DNA double-strand break (DSB) repair is impaired by RK-33

**Figure 9:**
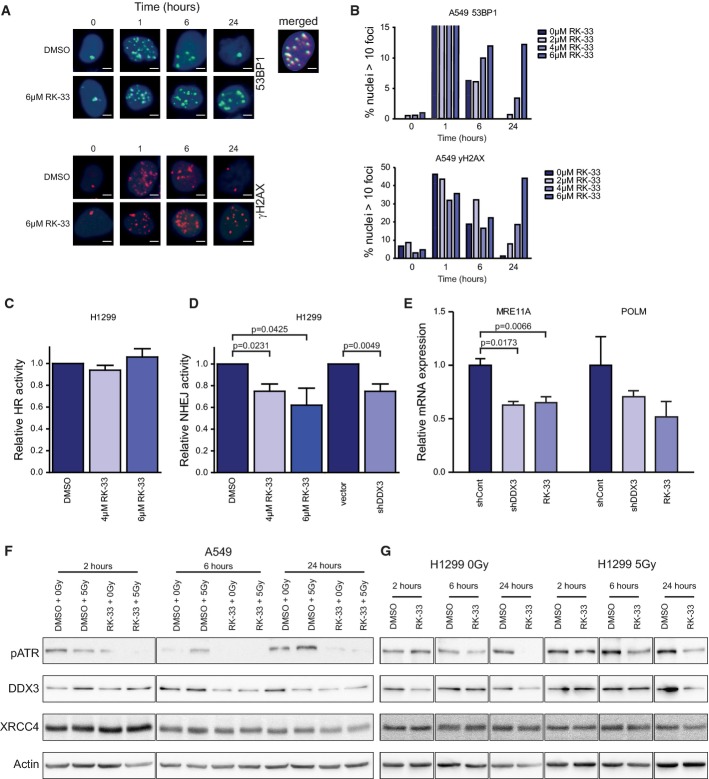
Effect of RK-33 on radiation-induced DNA damage

As RK-33 promoted radiation sensitization, we evaluated the DNA damage response following combination treatment with RK-33 and γ-radiation. Following γ-radiation, 53BP1 and γ-H2AX foci numbers increased within an hour and returned to pre-radiation foci numbers by 24 h. However, 53BP1 and γ-H2AX foci persisted 24 h post-RK-33 treatment, indicating reduced or delayed DNA repair (Fig9A). The extent of the impaired DNA damage repair was quantified by counting the number of severely damaged cells (> 10 foci per nucleus). This was determined for both 53BP1 foci (Fig9A, top panel) and γ-H2AX foci (Fig9A, bottom panel). Most cells were severely damaged 1 h after radiation, but a large reduction in 53BP1 and γ-H2AX foci after 6 h and normalization after 24 h, without significant cell death, indicated proficient DNA damage repair in untreated A549 cells. Importantly, when these cells were pre-treated with RK-33, DNA damage persisted well beyond 24 h (Fig9B).

A Immunofluorescence images showing 53BP1 and γH2AX foci in A549 cells after 2-Gy radiation and A549 cells pre-treated with 6 μM RK-33, 12 h before radiation. Overlap of 53BP1 and γH2AX is seen in the merged picture of the co-immunofluorescence staining. Scale bar is 2 μm.

B A549 cells were pre-treated with RK-33 and radiated with 2 Gy, and 53BP1 and γH2AX foci were counted as a measure of DNA damage. Cells with more than 10 foci 53BP1 or γH2AX were counted. More than 400 cells per sample were evaluated.

C H1299 cells stably transfected with a homologous recombination (HR) reporter construct were treated with RK-33. Reporter constructs expressed GFP, which was quantified by flow cytometry. Experiments were repeated three times.

D H1299 cells, containing a stable non-homologous end-joining (NHEJ) reporter construct, were treated with RK-33 and knockdown of DDX3. Reporter construct expressed GFP, which was quantified by flow cytometry. All experiments were repeated three times.

E Microarray results from MDA-MB-231 cells treated with RK-33 and shDDX3 were validated by qRT–PCR using NHEJ Mechanisms of DSBs Repair PrimePCR plates (Bio-Rad) and performed in biological triplicates.

F, G DNA repair-related proteins (ATR and XRCC4), DDX3, and actin were assessed by immunoblotting in A549 (F) and H1299 (G) cells. Cells were pretreated for 4 h with vehicle control or 6 μM RK-33 and then radiated with 0 or 5 Gy. Outlined boxes indicate spliced lanes. Data information: Significance was assessed by two-sided, paired *t*-test. Error bars represent SD. Source data are available online for this figure.

Next, we evaluated whether RK-33 could impair homologous recombination (HR) and non-homologous end joining (NHEJ), as these are the predominant DNA repair mechanisms in double-strand break repair (Valerie & Povirk, 2003). To determine this, we used two different stable cell lines with either a HR reporter construct (Chan *et al*, 2008) or a NHEJ reporter construct (Kriegs *et al*, 2010). After treatment with RK-33, we found no changes in HR activity but observed a significant 38% reduction of NHEJ (Fig9C and D). Moreover, we observed reduced NHEJ activity after knockdown of DDX3 (Fig9D). Our mRNA analysis indicated that this could be due to decreased expression of MRE11A and POLM, two genes shown to be important for NHEJ activity (Fig9E).

Next, we analyzed different proteins involved in NHEJ like ATR, and XRCC4 in RK-33-treated cells, with and without radiation. Treatment of A549 and H1299 cells with RK-33 and radiation caused a decrease of ATR but not XRCC4 (Fig9F and G). Also, there was no difference in two other DNA repair proteins, XRCC1 and Ku70. In conclusion, RK-33 impairs radiation-induced DNA damage repair by inhibiting NHEJ activity.

## Discussion

The concept of non-oncogene addiction postulates that certain non-oncogenic genes are critical for survival of cancer cells but are not required, to the same degree, for the viability of normal cells and are therefore attractive targets for cancer drugs (Solimini *et al*, 2007). One such gene is DDX3, a member of the RNA helicase family, which we have shown to be dysregulated in breast cancer cell lines, up-regulated by HIF-1α, and involved in cancer maintenance and metastasis (Botlagunta *et al*, 2008, 2011; Bol *et al*, 2013). Furthermore, we have published that DDX3 is an essential component for cellular proliferation and a decrease in its functional activity can lead to cellular stasis. Collectively, the function of DDX3 in cellular biogenesis, RNA metabolism, and translation has driven an interest to identify small molecule inhibitors of DDX3. In this paper, we show that abrogating DDX3 function leads to potent radiation sensitization in lung cancer, through inhibition of NHEJ and Wnt signaling.

Besides the concept that DDX3 is essential for the cellular stress response, proliferation, and evasion of apoptosis, there are some data to indicate that DDX3 may have alternative functions and potential tumor suppressive functions (Chang *et al*, 2005; Wu *et al*, 2014). However, by mining the COSMIC database, we found only 7.7% of genetic abnormalities of the DDX3 gene typical for tumor suppressor genes (nonsense mutations, deletions or loss of heterozygosity), whereas 87.2% of DDX3 genetic abnormalities are more typical for a gain of function (substitution missense mutations). Nevertheless, it is possible that the functions of DDX3 are organ specific and may require cell-specific co-factors to determine its effect. Interestingly, a recent paper examined the role of Wnt/β-catenin signaling in *Xenopus* and *C. elegans* development and concluded that DDX3 is required for Wnt signaling (Cruciat *et al*, 2013).

Lung cancer patients are often treated with radiation therapy. Higher doses per fraction radiation can result in better local control but, dependent on tumor location or when re-treatment is needed, can be limited by toxicity (Senthi *et al*, 2012). In our combination studies with RK-33 and radiation, we saw a synergistic effect *in vitro* and greater than additive effects in two preclinical models of lung cancer. However, radiation sensitization of RK-33 in combination with a fractionated radiation schedule had only limited effect *in vivo*. Given the radiosensitization we observed *in vitro* by clonogenic assays with standard doses of radiation (< 3 Gy), we propose that limited effect *in vivo* with standard fractionated radiation could be due to the relatively infrequent injections of RK-33 in relation to radiation treatments.

The combination effect of RK-33 and radiation *in vitro* and *in vivo* was apparent in the reduction of DNA damage repair following radiation and RK-33 treatment. Mechanistically, Wnt/β-catenin signaling can mediate radiation resistance (Woodward *et al*, 2007; Zhang *et al*, 2010) but its precise mechanism is yet to be identified. To explore this, we assessed two of the most important repair mechanisms of DSBs: NHEJ and HR (Valerie & Povirk, 2003) and found RK-33 to inhibit NHEJ activity but not HR. The impairment of NHEJ results in reduced DSB repair, leading to genomic toxicity and thus potentiating cell death in DDX3-dependent cells. Although not all molecular interactions of DDX3 in cellular biogenesis and adaption are known, it is clear that DDX3 is an essential component of Wnt signaling and is pivotal for the maintenance of the tumorigenic state. Also, as RK-33 causes G1 arrest and NHEJ is the predominant pathway for DSB repair in this phase of the cell cycle, this could result in profound radiosensitization. Thus, targeting the non-oncogene addiction of lung cancer cells to DDX3 by RK-33 will shift their delicate balance toward tumor death.

In summary, DDX3 is a hallmark of aggressive lung cancer and serves as a promising target for radioresistant lung cancer. Moreover, RK-33 as a radiation sensitizer, because of its specificity to cancer cells and mild side effects, could lead to increased lung cancer patient survival and better quality of life.

## Materials and Methods

### Clinical samples

Representative paraffin-embedded tissue blocks of 95 lung cancer patients were taken from the archive of the Department of Pathology of the University Medical Center in Utrecht and routinely processed into a tissue microarray (TMA). Intensity of cytoplasmic DDX3 was scored semi-quantitatively by two independent pathologists (*r* = 0.743). Survival statistics were obtained from the Comprehensive Cancer Center, The Netherlands (IKNL). Use of anonymous or coded left over material for scientific purposes is part of the standard treatment contract with patients in the UMCU (van Diest, 2002).

### Immunohistochemistry

Sections of 4 μm were cut, mounted on SuperFrost slides (Menzel & Glaeser, Brunswick, Germany), deparaffinized, and rehydrated. Endogenous peroxidase was then blocked for 15 min with a buffer solution containing 0.3% hydrogen peroxide. Antigens were retrieved by boiling for 30 min in 10 mM citrate buffer (pH 6.0) and cooled and washed in PBS. Slides were subsequently incubated in a humidified chamber for 1 h with polyclonal rabbit anti-DDX3 (Angus *et al*, 2010) diluted 1:1,000. Subsequently, sections were washed in PBS and incubated for 30 min with secondary antibodies (Brightvision, Immunologic, Duiven, The Netherlands), washed with PBS and developed with diaminobenzidine. Slides were counterstained with hematoxylin, dehydrated, and cover-slipped. Appropriate positive (validated breast cancer sample, thymus) and negative controls (normal lung tissue, normal liver tissue) were used throughout.

### Scoring of immunohistochemistry

Scoring was done by two experienced pathologists (Paul J. van Diest and Stefan Willems). Intensity of cytoplasmic DDX3 was scored semi-quantitatively from 0 to 3, H-scores were determined, and percentages of cells with nuclear DDX3 expression were estimated. Out of three cores from the same patient, the maximum cytoplasmic DDX3 score was used for further analysis.

DDX3 scores 1 and 2 were grouped as low DDX3 expression and evaluated against high DDX3 expression (scores 3). Cytoplasmic expression of DDX3 was uniform within a single tumor (Fig1) as shown by similar H-scores and intensity scores of DDX3 expression. Only intensity scores are shown.

### Statistics

Expression levels of DDX3 were compared between different histological subtypes by chi-square test or Fisher's exact test, whichever was appropriate. The relation between DDX3 and the mortality of lung cancer patients was assessed by Kaplan–Meier curves and compared with the log-rank test. The relation between DDX3, mortality, and clinically relevant variables related to aggressiveness (tumor size, histological type, and grade) were assessed by univariate cox regression analysis. Also, a multivariate regression model was made in which tumor size was excluded as it has a univariate *P*-value > 0.2 and made the multivariate regression model less predictive.

All statistical analyses were carried out with SPSS 17.0 for Windows (SPSS Inc., Chicago, IL, USA), regarding two-sided *P*-values below 0.05 as significant.

### Helicase assay

The RNA helicase assay was performed as described earlier (Sengoku *et al*, 2006) with a few modifications. Briefly, RNA oligomers R41 (unlabeled 41 nt) 5′-CGAAAGCACCGUAAACG-AAAACUAGCACCGUAAAGCAAGCU-3′ and R13 (FAM-labeled 13 nt) 5′-CGUUUACGGUGCU-3′ were annealed to form radiolabeled RNA duplexes and incubated with R13C (unlabeled 13-nt quencher RNA complementary to R13) 5′-AGCACCGUAAACG-3′, along with Ded1p protein (80 nM), 100 μM ATP, and RNA inhibitor RNasin (Promega, Madison, WI, USA) in reaction buffer containing 50 mM Tris, pH 7.8, 2 mM MgCl_2_, 3% glycerol, and 1 mM DTT for various times at room temperature. Reactions were stopped after 30-min incubation at room temperature with stop solution containing proteinase K (1 mg/ml) and SDS (0.6%). Reactions were loaded on acrylamide gels and bands visualized using a Typhoon scanner (GE Healthcare, Piscataway, NJ, USA).

### Docking

The 3D structure of RK-33 was built and energy-minimized with ChemBio 3D Ultra 12.0 Suite (Cambridge Soft, USA) using MMFF94 force field. DDX3 structure was retrieved from NCBI with PDB ID 2I4I. Using the algorithm Iterated Local Search global optimizer with scoring function similar to X-Score tuned by PDBbind, we docked the RK-33 to the ATP-binding domain of DDX3 with Autodock Vina program (Trott & Olson, 2010). Figures were prepared with PyMOL (http://www.pymol.org/).

### Proliferation and viability assay

Proliferation was determined by counting cancer cells over time. Cells were seeded at 7.5 × 10^4^ cells/well and counted daily with a TC10 automated cell counter (Bio-Rad, Hercules, CA, USA) at least three times. Viability of cancer cells was determined by MTS assay (CellTiter 96® AQueous One Solution, Promega, Madison, WI, USA) as described by the manufacturer. Briefly, 800–2,000 cells were allowed to attach overnight in a 96-well plate, treated with different concentrations of RK-33 and kept for 72 h. MTS reagent was kept for 2 h. Each experiment was repeated a minimum of three independent times.

### NCI-60 assay

The Developmental Therapeutics Program (DTP) of the NCI offers a service in which the NCI-60, a set of 60 human tumor cell lines derived from various tissues of origin, are tested for sensitivity to selected compounds. RK-33 was selected and tested twice at the 5 dose-response (http://dtp.nci.nih.gov/branches/btb/ivclsp.html). We downloaded GI_50_ doses for 102 FDA-approved cancer drugs in the NCI60 cell lines from the NCI/NIH Developmental Therapeutics Project. (http://dtp.nci.nih.gov/index.html). The values for the FDA-approved drugs as well as for RK-33 were transformed according to the formula D = −log10 (GI_50_) so that large values of D indicate sensitivity to the given drug. Spearman rank-based correlations of GI_50_ doses were used to measure associations between drugs. An edge is drawn between two drugs if the correlation is at least 0.60 (Spearman's rho).

### Microarrays and analysis

Healthy, 60–70% confluent MDA-MB-231 cells were transduced with shDDX3 lentivirus particles. Knockdown of DDX3 expression was confirmed both by qRT–PCR and immunoblotting. MDA-MB-231 cells were treated with RK-33 (7.5 μM) for 12 h and harvested for RNA using RNeasy columns (Qiagen, Germantown, MD, USA). All treatments were performed in triplicates. Microarray experiments were performed at the Johns Hopkins Deep Sequencing and Microarray Core using Human Gene 1.0 ST arrays (Affymetrix, Santa Clara, CA, USA). Data analyses were performed using BRB-ArrayTools developed by Dr. Richard Simon and the BRB-ArrayTools Development Team (http://linus.nci.nih.gov/BRB-ArrayTools.html). Briefly, genes showing minimal variation across the set of arrays were excluded from the analysis. Genes whose expression differed by at least 1.5-fold from the median in at least 20% of the arrays were retained. We identified genes that were differentially expressed among the two classes using a random-variance *t*-test, which does not assume equal variance (Wright & Simon, 2003). Genes were considered statistically significant if their *P*-value was less than 0.001.

The evaluation of which Gene Ontology (GO) classes are differentially expressed between pre- and post-treatment samples was performed using a functional class scoring analysis as described (Pavlidis *et al*, 2004). Functional class scoring is a more powerful method of identifying differentially expressed gene classes than the more common over-representation analysis or annotation of gene lists based on individually analyzed genes. We considered a GO category significantly differentially regulated if the significance level was less than 0.01 in either the Fisher (LS) or Kolmogorov–Smirnov (KS) statistic test.

### TCF reporter assay

Transcriptional activity of TCF4 was assayed using the dual luciferase assay (Promega, Madison, WI, USA) according to the manufacturer's instructions. Briefly, 1.5 × 10^4^ A549 or H1299 cells were transfected with 500 ng of TOP-FLASH and control FOP-FLASH constructs along with 50 ng of phRL *Renilla* constructs as transfection controls as well as with 500 ng β-catenin constructs when indicated. Cells were cultured for 24 h and then lysed in passive lysis buffer. Luminescence was detected using a luminometer (Berthold Sirius, Oak Ridge, TN, USA). Relative TCF4 promoter activity was calculated by dividing firefly luminescence by *Renilla* luminescence, and then normalized TOP-FLASH was divided by normalized FOP-FLASH, which was finally normalized to vector or DMSO control cells. All experiments were repeated three times, and differences were assessed by the paired *t*-test.

### Co-IP

Co-immunoprecipitation was carried out using antibodies crosslinked to protein A/G magnetic beads (Thermo Fisher, Rockford, IL, USA). Briefly, 5–10 μg antibodies were bound to twice pre-washed beads for 15 min at room temperature. The antibody–beads complex was washed thrice and cross-linked with disuccinimidyl suberate for 30 min at room temperature. The cross-linked antibody–beads complex was washed five times to elute non-specific and non-cross-linked antibodies. Subsequently, cell lysates from A549 and H1299 cells were incubated overnight with antibody cross-linked beads at 4°C on a rotator. The complex was washed twice and eluted in buffer (pH 2.0) for 5 min at room temperature. The eluate was neutralized with buffer (pH 8.5), loaded on an SDS–polyacrylamide gel, and visualized by immunoblotting.

### Biotin bead assay

Two plates of 70% confluent cells were lysed with IP lysis buffer (Tris–HCl (pH 7.4) 25 mM, NaCl 150 mM, EDTA 1 mM, NP-40 1%, glycerol 5%, protease inhibitor). Cell lysate or DDX3 protein (S2-G582) was incubated with 10 μM biotin (ctrl), SK-153 or AK-298 (biotin-linked RK-33) for 2 h at 4°C. Streptavidin beads were added and incubated for 1 h at room temperature. The biotin-bead–RK-33 complex was extracted with a magnetic stand and washed seven times with PBS. Proteins were analyzed by immunoblot.

### Cell lines

All cell lines were obtained from the American Type Culture Collection and maintained as recommended (ATCC, Manassas, VA, USA). DDX3 shRNA lentiviral constructs were described earlier (Botlagunta *et al*, 2011). Briefly, this construct has a U6 promoter driving DDX3 shRNA and a PGK promoter driving EGFP. Controls used were empty vector not containing shRNA. Cells for the NHEJ assay, H1299.EJ, were a kind gift of Professor Ekkehard Dikomey (University of Hamburg, Hamburg, Germany). Cells for the homologous recombination assay, H1299-DR-GFP, were a kind gift of Professor Robert Bristow (University of Toronto, Ontario, Canada). For the respiratory studies, highly aggressive proliferating immortalized (HAPI) cells were cultured under 95% air/5% CO_2_ in Dulbecco's modified Eagle's medium (DMEM) supplemented with 10% fetal bovine serum, L-glutamine (2 mM), penicillin (100 IU/ml), and streptomycin (100 μg/ml) at 37°C.

### Biodistribution and *in vitro* metabolism of RK-33

RK-33 was quantitated in plasma, tissue, or microsomal preparations. RK-33 metabolism studies were conducted in a 100-mM sodium-potassium phosphate buffer (pH 7.4) containing 20 mg/ml human or mouse liver microsomes (BD Gentest, Woburn, MA) and 5 mM of RK-33. Incubations were performed at 37°C in the presence or absence of NADPH-generating system to control for native enzyme activities. Tissue homogenates were prepared at a concentration of 200 mg/ml in PBS and further diluted 1:10 in plasma prior to extraction. RK-33 (100 μl of sample) was extracted with 300 μl of acetonitrile. After centrifugation, the supernatant was injected into the LC-MS/MS system consisting of a Waters Acquity UPLCTM system coupled to an AB SCIEX Triple Quad TM 5500 mass spectrometer. Separation of the analyte from potentially interfering material was achieved at ambient temperature using Waters XTerra ODS column (50 × 2.1 mm i.d., 3 μm). The mobile phase used was composed of acetonitrile–water (60:40, v/v) containing 0.1% formic acid and was delivered isocratically at a flow rate of 0.2 ml/min. The column effluent was monitored by the mass spectrometer that was equipped with an electrospray interface, operated in a positive mode. The following transitions were monitored: 429.0 > 121.0 for RK-33 and 301.10 > 255.10 for the IS. Results were assessed qualitatively comparing the average area ratio of RK-33 at 0 h to area ratio at 0.5 h and 1 h for both mouse and human liver microsomes. The same samples were injected again scanning Q1 only to get a full-scan spectrum for m/z ratios between 300 and 500 to determine any other peaks of interest. Calibration curves for RK-33 were computed using the area ratio by using linear regression with a 1/x weighting function over the range of 5–1,000 ng/ml with dilutions of up to 1:100 (v:v).

### Animals

Mice were housed in groups of five per cage with free access to food and water, under controlled light/dark cycles, in facilities with regulated temperature and humidity. Mice were randomly assigned to different experimental groups, and researchers conducting the experiments were blind to experimental condition. All procedures were approved by the Institutional Animal Care and Use Committee of The Johns Hopkins University.

Inducible Twist1/Kras^G12D^ transgenic mice in the FVB/N inbred background were of the genotype: CCSP-rtTA/tetO-KrasG12D/Twist1-tetO-luc (Tran *et al*, 2012). All the mice were weaned 3–4 weeks of age and then placed on dox at 4–6 weeks of age. Mice were treated with similar levels of tumor burden per micro-CT and were typically 20–30 weeks old. Mice were treated as in Fig6C and H. Each group consisted of mice, of which multiple index tumors were followed in time by micro-CT and quantified by a board-certified radiation oncologist (JZ) in a blinded fashion. Bi-dimensional measurements were made on tumors using serial examinations and tumor volumes calculated using the equation Volume = π/6 × 1.65 (length × width) × 3/2. Volumes were normalized to the starting volume, *t* = 0 before treatment. For 15-Gy SABR experiments, the breakdown of animal usage is (1) RT = 3 female mice, (2) RK-33 = 3 females and 1 male mice, (3) carboplatin = 3 male mice, (4) RT + carboplatin = 3 female mice, and (5) RT + RK-33 = 5 female mice. For the 3-Gy XRT experiments, animals used were (1) RT = 3 female and three male mice and (2) RT + RK-33 = four female and three male mice.

Athymic NCr-nu/nu female mice 4–5 weeks old were purchased from NCI Frederick. A total of 1 × 10^6^ A549 cells were injected in the tail vein of ten mice. Treatment (five animals per group) was started 4 weeks after inoculation of tumor cells. Since tumor volumes could not be determined by micro-CT, tumor burden was established at necropsy by histopathology, 8 weeks after inoculation of tumor cells. All five lung lobes were paraffin-embedded and mid-lung sections were stained with H&E. Slides were digitalized; lung and tumor surface area was calculated with ImageScope (Aperio Technologies, Vista, CA, USA), as tumor burden = tumor surface/lung surface.

### Radiation therapy

Cells were irradiated at room temperature with 0.5 Gy/min to the desired dose using a Gammacell 40 ^137^Cs irradiator. For *in vivo* experiments, mice were treated using the Small Animal Radiation Research Platform (Wong *et al*, 2008). The tumors were irradiated with a circular beam of 1 cm diameter.

### Micro-CT image analysis

The mice underwent micro-CT imaging by use of the Small Animal Radiation Research Platform (SARRP) irradiator described earlier (Wong *et al*, 2008). The uncollimated primary beam was used for imaging. A total of 1,800 projections were acquired at approximately 0.2 angular increments. The Feldkamp CBCT algorithm was used for reconstruction. Tumor volumes were quantified from the CT dataset with Pinnacle3 software v.8.1y (Philips Inc, Madison, WI) as described previously (Tran *et al*, 2011; Zeng *et al*, 2013). Briefly, we viewed 2D images in sagittal, coronal, and transverse views to detect tumors and track them over time. Matching tumors across time points was performed manually by simultaneous viewing of the serial data. This analysis was performed by a board-certified radiation oncologist. This volume information was then used for analysis of the temporal changes in lung tumor nodules. Micro-CT imaging has been shown to correlate with the number and volume of murine lung tumors found on necropsy (Cody *et al*, 2005).

### SA-β-gal staining

Cells were washed twice with phosphate-buffered saline (PBS) and then fixed with 3% formaldehyde for 5 min. The cells were then washed with PBS and incubated at 37°C overnight with staining solution (30 mM citric acid sodium phosphate, pH 6.0, 1 mg 5-bromo-4-chloro-3-isolyl-b-D-galactoside [X-gal, Fisher], 5 mM potassium ferricyanide, 5 mM potassium ferrocyanide, 150 mM NaCl, 2 mM MgCl_2_). After incubation, cells were washed with PBS and viewed with bright-field microscopy.

### Colony-forming assay

Cells were counted, plated, and allowed to attach overnight. RK-33 at specified doses was added 4 h before radiation was delivered. Colonies were stained and counted approximately 10 days after irradiation. Surviving fraction was calculated by dividing the number of colonies formed by the number of cells plated times the plating efficiency. Curves were fitted with a quadratic polynomial equation. *P*-values were determined by the extra sum of square *F*-test.

### Cell cycle analysis

A549 and H1299 cells were plated at 1 × 10^5^ cells per well of a 6-well plate. Cell cycle was carried out as previously described (Vesuna *et al*, 2012). Briefly, cells were trypsinized 24 h after plating and fixed in 70% ethanol overnight at −20°C. Fixed cells were washed with PBS and resuspended in DNA-staining solution (5 μg/ml propidium iodide, 0.5 mg/ml RNase A) for 1 h at room temperature. Cell cycle acquisition was performed on a FACScan I or FACSCalibur instrument (BD Biosciences, San Jose, CA, USA). Independent experiments were repeated three times. Data were analyzed using FlowJo software (Tree Star Inc., Ashland, OR, USA).

### Immunofluorescence

A549 cells were allowed to attach overnight in chamber slides. For DDR experiments (Fig6A), cells were incubated with RK-33 for 12 h and then radiated with 2 Gy. After 0, 1, 6, and 24 h, cells were fixed and stained. Cells were fixed for 15 min in 4% formalin, washed with PBS, permeabilized (PBS containing 0.2% Triton X-100) for 5 min, and blocked for 30 min with 10% goat serum. Cells were incubated with anti β-catenin (1:1,000, mAb C7082, Clone 6F9, Sigma-Aldrich), DDX3 (1:1,000, pAb) (Angus *et al*, 2010), 53BP1 (1:1,000, pAb, NB100-304, Novus Biologicals), or γH2AX (1:1,600, mAb, DAM1782241, Millipore) antibodies in 0.5% BSA/PBS for 1 h. Next, cells were washed with PBS and incubated with secondary antibodies, CY3 (1:200, goat anti-mouse, 115-165-071, Jackson Immuno Research) or Alexa fluor 488 (1:200, goat anti-rabbit, A11034, Invitrogen) for 1 h. Cells were washed, nuclei-stained with 4′,6-diamidino-2-phenylindole (DAPI), and cover-slipped. Photographs were taken with Nikon Eclipse 80i fluorescence microscope using a CoolSnap ES camera (Roper scientific, Sarasota, FL, USA).

### Immunoblotting

Cell lysate was prepared with TB buffer method. For immunoblotting, ~25 μg protein was loaded on 10% SDS–PAGE gels. Following gel electrophoresis, proteins were transferred onto PVDF membrane, blocked with 5% milk, and probed with primary antibodies against DDX3 (mAb) (Angus *et al*, 2010), actin (A5441, Sigma-Aldrich), Cyclin D1 (2926S, Cell Signaling), Erk1/2 (4695S, Cell Signaling), pErk1/2 (4370S, Cell Signaling), β-catenin (pAb C2206, Sigma-Aldrich), pATR (2853, Cell Signaling), pATM (Cell Signaling), XRCC4 (ab145, Abcam), DDX5 (pab204, EMD Millipore), DDX17 (Bethyl), and appropriate secondary antibodies. The blots were developed with clarity Western ECL (Bio-Rad, Hercules, CA, USA) and imaged with G:BOX Chemi XR5 (Syngene, Frederick, MD, USA).

### Quantitative reverse-transcription polymerase chain reaction

RNA was extracted according to the manufacturer's instructions (Qiagen, Valencia, CA, USA), and cDNA was manufactured using qScript cDNA synthesis kit (Quanta BioSciences, Gaithersburg, MD, USA), followed by qPCR using SYBR green (Quanta BioSciences) on an iCycler5 (Bio-Rad, Hercules, CA, USA). Amplification of 36B4, a housekeeping gene, was used for normalizing gene expression values. Primer sequences were as follows: DDX3 F 5′-GGAGGAAGTACAGC CAGCAAAG-3′, DDX3 R 5′-CTGCCAATGCCATCGTAATCACTC-3′, Axin-2 F 5′-TCAAGTGCAAACTTTCGCCAACC-3′, Axin-2 R 5′-TAGCCAGAACCTATGTGATAAGG-3′, c-Myc F 5′-CGTCTCCACACATCAGCACAA-3′, c-Myc R 5′-CACTGTCCAACTTGACCCTCTTG-3′, Cyclin D1 F 5′-GGCGGAGGAGAACAAACAGA-3′, Cyclin D1 R 5′-TGGCACAGAGGGCAACGA-3′.

### NHEJ and HR reporter cell lines

NHEJ was assessed by the functional reporter system H1299-EJ, provided by E Dikomey. In brief, if untreated, these reporter cells do not express functional GFP because of an artificial off frame start codon, which is flanked by two I-SceI restriction sites. When these sites are incised by I-SceI, the artificial start codon is removed creating a DSB. Repair of this DSB by NHEJ restores GFP expression, so that cells capable for NHEJ can be identified by their GFP fluorescence intensity (Kriegs *et al*, 2010). H1299.EJ cells were plated in a 6-well plate (1 × 10^5^ cell/well) and treated with 4–6 μM RK-33. The following day, cells were transfected (in duplicates) with 1–2 μg of pSceI or pEGFP construct using *Trans*IT-LT1 (Mirus, Madison, WI, USA) in serum-free media (Opti-MEM, Invitrogen, Carlsbad, CA, USA). Media was replaced after 4–6 h with complete media. Subsequently, cells were harvested by trypsinization, washed in PBS, and resuspended in 0.5 ml PBS. NHEJ activity was measured on a FACScan I instrument and analyzed on FlowJo software. Recombination frequency was calculated as ((% pSceI − % negative control) / % pEGFP). H1299-DR-GFP cells, provided by RG Bristow, were used to measure homologous recombination similarly as described above (Chan *et al*, 2008).

### XF24 microplate-based respirometry

Oxygen consumption measurements were performed using an XF24 Extracellular Flux Analyzer (Seahorse Bioscience, Billerica, MA) as previously described (Wu *et al*, 2007; Clerc & Polster, 2012). HAPI cells were plated at a density of 4 × 10^4^ cells per well to achieve ~85% confluence at the time of assay (16–24 h after plating). XF24 assay medium consisted of 120 mM NaCl, 3.5 mM KCl, 1.3 mM CaCl_2_, 0.4 mM KH_2_PO_4_, 1 mM MgCl_2_, 5 mM HEPES, 15 mM glucose, and 4 mg/ml fatty acid-free bovine serum albumin, pH 7.4. Cells were incubated in glucose- and bovine serum albumin-free XF24 assay medium supplemented with 2-deoxyglucose (50 mM) and pyruvate (10 mM) with or without experimental treatments for 1 h at 37°C. The ATP bioluminescent somatic cell assay kit (Sigma-Aldrich) was employed for the determination of ATP levels by luminescence using a FLUOstar OPTIMA multimodal plate reader (BMG LABTECH, Inc., Cary, NC). Total ATP content was normalized to cellular protein. One-way analysis of variance (ANOVA) was used to evaluate statistical significance, and Tukey's *post hoc* analysis was used to compare individual groups. SigmaPlot 12.0 (Systat Software, Inc., San Jose, CA) was used for the analysis.

**The paper explained**

**Problem**

Targeting oncogenes for cancer therapy, although practical, has its limitations with regard to therapeutic efficacy and toxicity. Interest in identifying and targeting non-oncogene addiction is gaining momentum due to the potential of broad-based applications for treatment of different cancers. Recent studies have indicated that targeting DNA repair pathways may enhance treatment efficacy and reduce side effects. Moreover, there is a drive to identify other non-oncogene addiction targets that will perturb multiple pathways in cancer cells with the end result of achieving higher survival rate and increasing the quality of life. In this study, we explore the functional utility of targeting a RNA helicase gene, DDX3, which is a non-oncogene addiction gene that is essential to maintain cancer cell survival under increased cellular stress associated with the tumorigenic state.

**Results**

In this study, we identified a RNA helicase, DDX3, which is overexpressed in lung cancer and is associated with lower survival in lung cancer patients. Importantly, knockdown of DDX3 in highly aggressive lung cancer cell lines (H1299 and A549) lowered their colony-forming abilities. In our efforts to abrogate DDX3 functions *in vivo*, we synthesized a small molecule inhibitor, RK-33, which was designed to bind to the nucleotide-binding site within the DDX3 protein and abrogate its functionality. We demonstrated that RK-33 was able to induce cell death in aggressive lung cancer cell lines and not in normal cells. Importantly, this small molecule showed no toxicity, at the therapeutic dose, in animal experiments. Also, RK-33 caused cell cycle arrest, induced apoptosis, and promoted radiation sensitization in DDX3-overexpressing cells. Notably, RK-33 in combination with radiation induced tumor regression in multiple mouse models of lung cancer. Mechanistically, RK-33 inhibited non-homologous end joining and impaired Wnt signaling by disrupting the DDX3–β-catenin axis.

**Impact**

The identification of DDX3 as an independent marker of lung cancer biogenesis and the ability to target tumor cells with RK-33 and radiation will provide a targeted chemotherapy option for treating lung cancer. Also, the combination with radiation will exhibit increased efficacy, accelerate treatment response, and reduce overall cost. As RK-33 showed no toxicity in animals, we expect to translate the preclinical data into clinics in an accelerated manner. Importantly, it will provide new avenues to increase the therapeutic effect of radiation and reduce potential side effects and increase quality of life.

## Supporting Information

Supplementary Figure Legends

Review Process File
