# Supplementary figures and images for: Targeting DDX3 with a small molecule inhibitor for lung cancer therapy
Source: EMBO Mol Med. 2015 Mar 27;7(5):648–69. doi: 10.15252/emmm.201404368 (PMC4492822; doi:10.15252/emmm.201404368)

**A**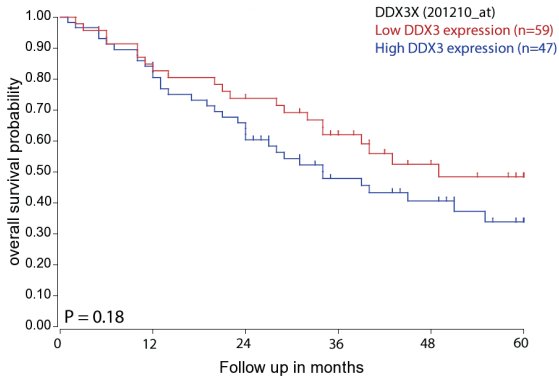**B**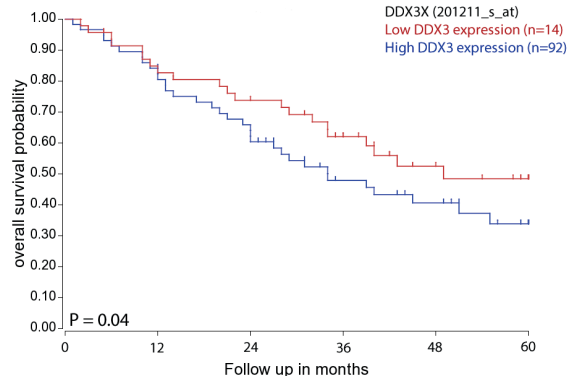**C**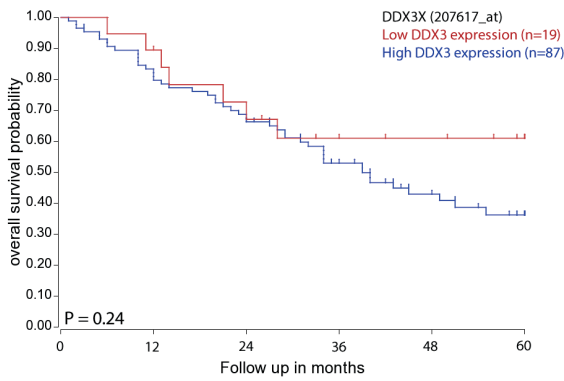**D**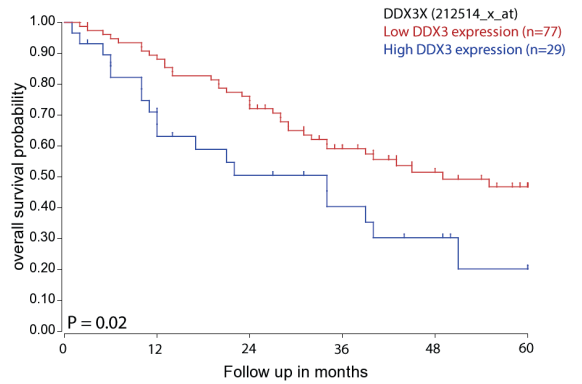**E**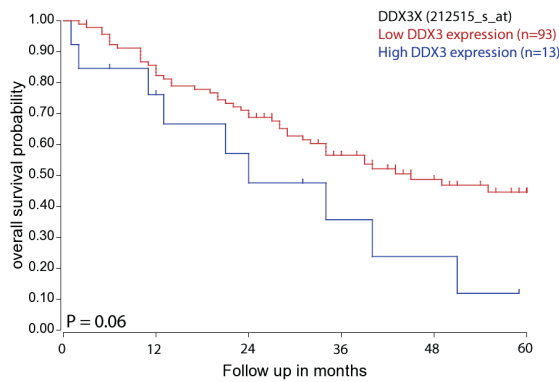**F**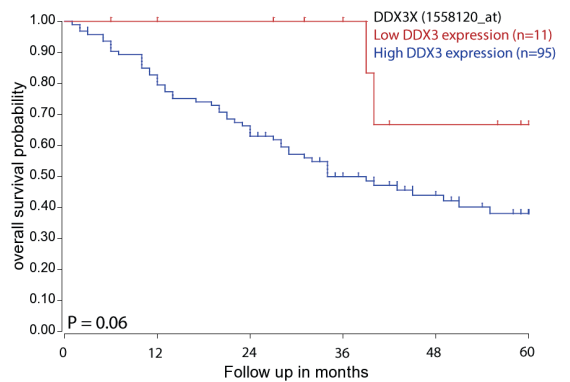

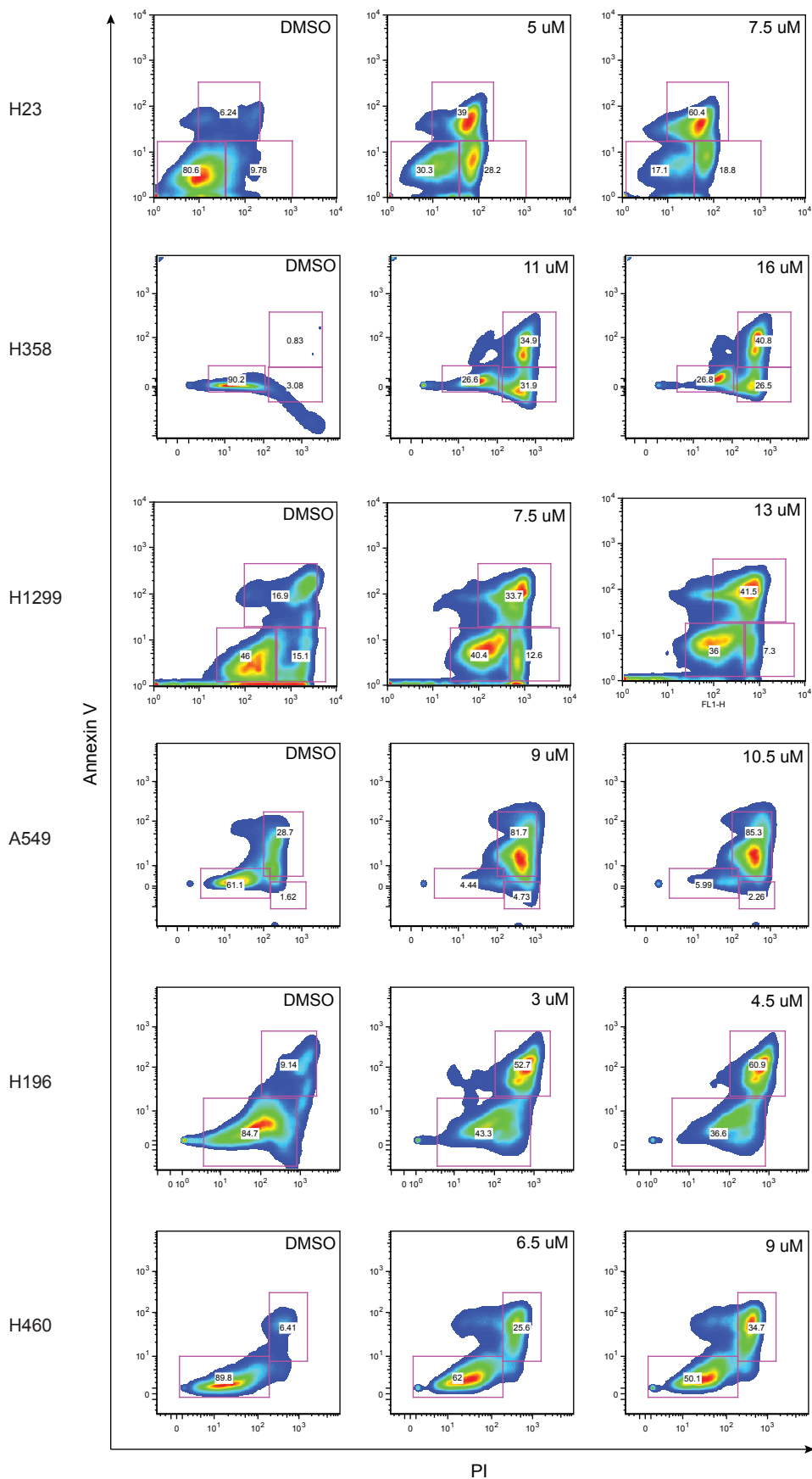

**A**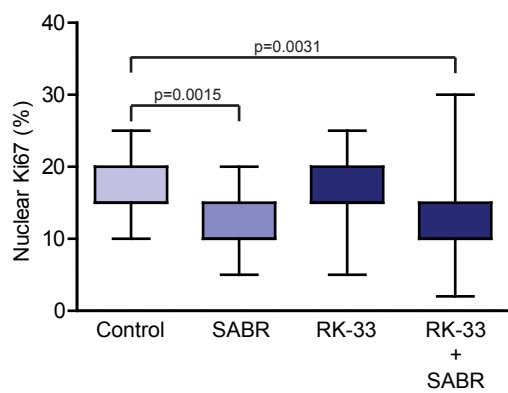**B**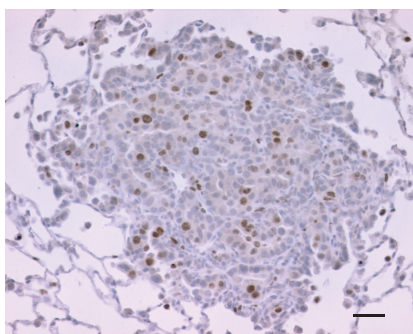**C**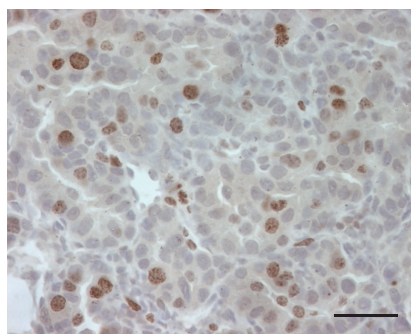**D**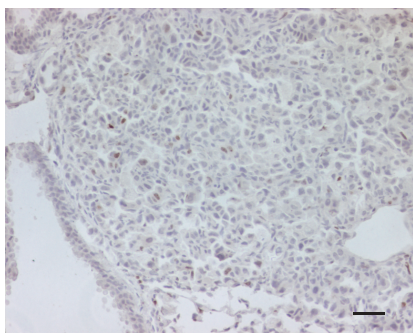**E**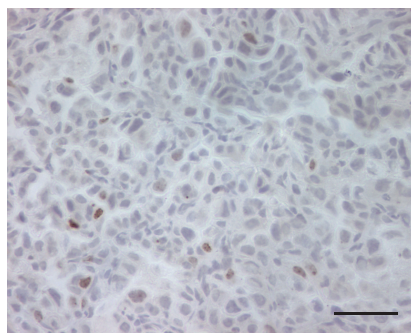

Supplement: Supplementary file 1 [file emmm0007-0648-sd1.pdf]

Figure 1

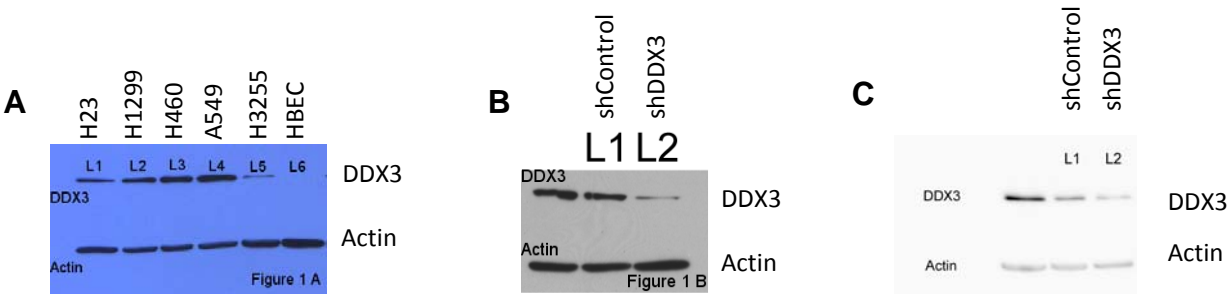

Supplement: Supplementary file 4 [file emmm0007-0648-sd4.pdf]

Figure 2

F

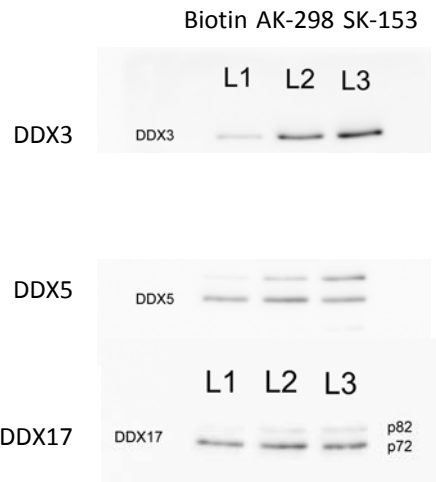

H

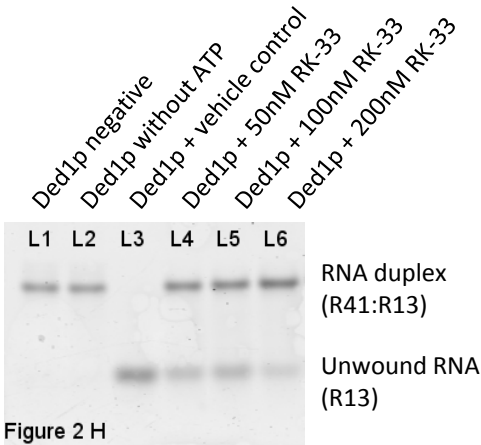

Supplement: Supplementary file 5 [file emmm0007-0648-sd5.pdf]

Figure 7

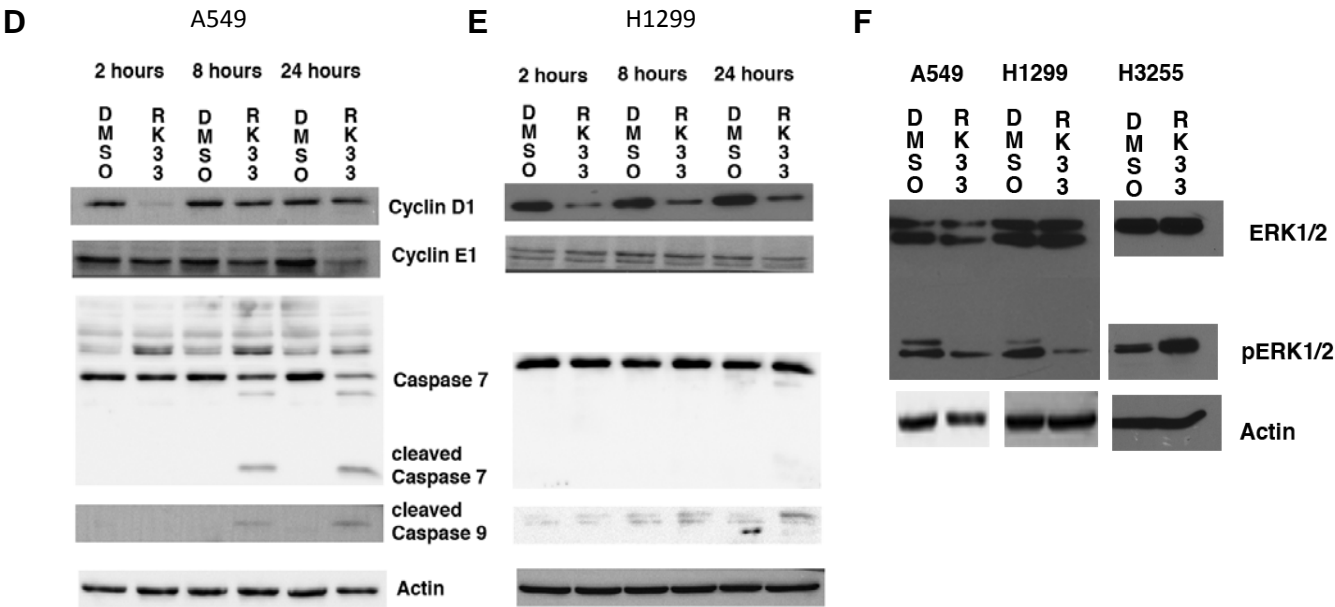

Supplement: Supplementary file 6 [file emmm0007-0648-sd6.pdf]

Figure 8

B

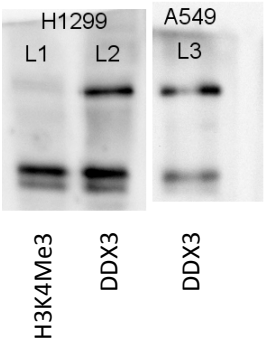

C

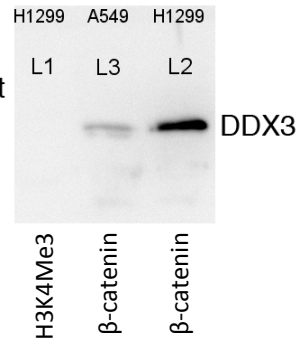

Supplement: Supplementary file 7 [file emmm0007-0648-sd7.pdf]

Figure 9

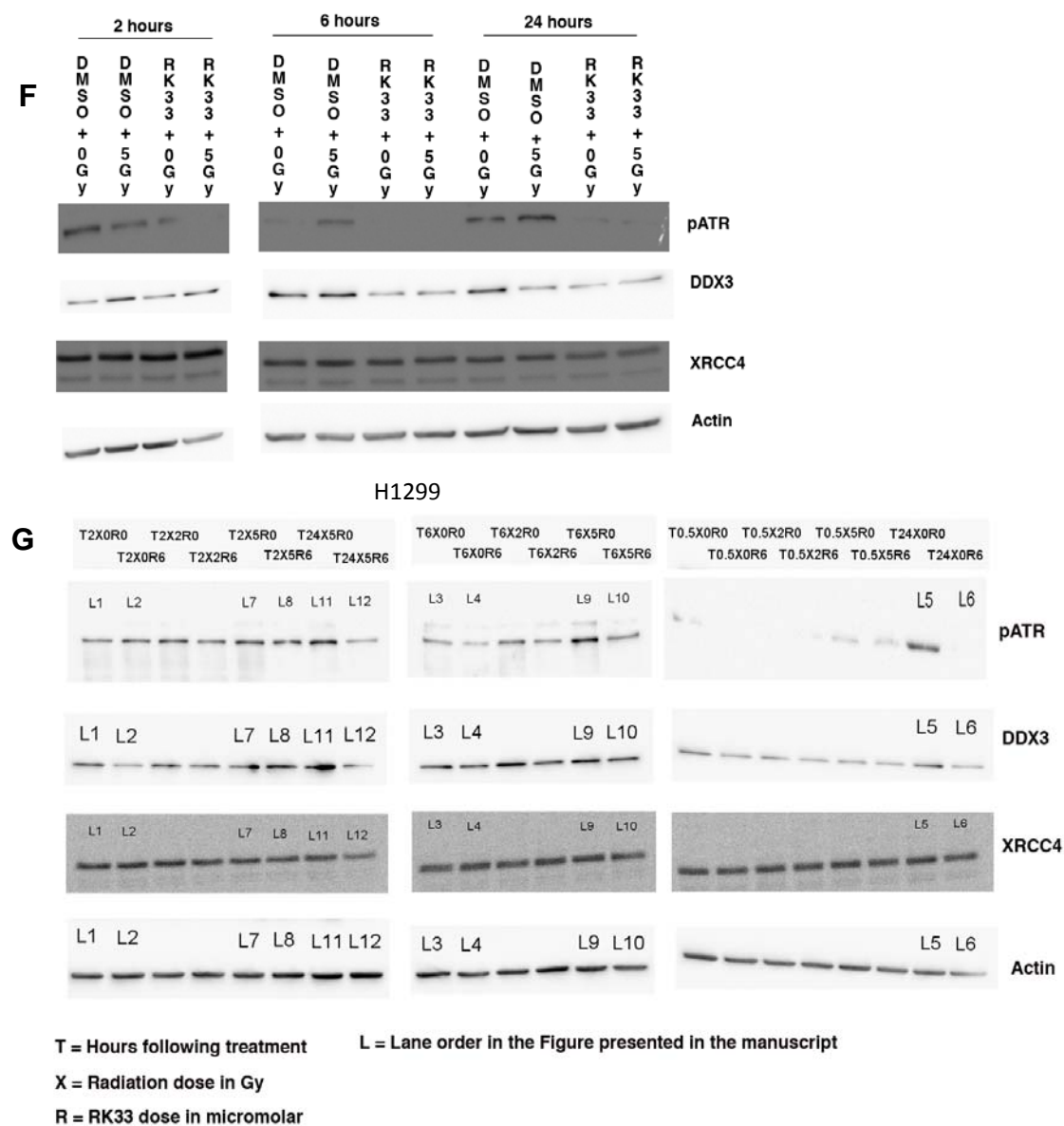

Supplement: Supplementary file 8 [file emmm0007-0648-sd8.pdf]
