# Supplementary material for: Targeting DDX3 with a small molecule inhibitor for lung cancer therapy
Source: EMBO Mol Med. 2015 Mar 27;7(5):648–69. doi: 10.15252/emmm.201404368 (PMC4492822; doi:10.15252/emmm.201404368)
Supplement: Supplementary file 2 [file emmm0007-0648-sd2.docx]

**Figure S1. DDX3 mRNA expression and survival in an independent NSCLC dataset**

Kaplan-Meier curves for six DDX3 probes (A-F) on an Affymetrix chip in a dataset with 114 NSCLC cases. Eight cases were excluded from the analysis because survival data was not available. P-values were calculated with the log-rank test.

**Figure S2.** **Apoptosis in RK-33 treated cells**

Histograms depicting early apoptosis (Annexin V positive) and late apoptosis (PI positive) in cells treated with the indicated amounts of RK-33. Cells were either treated with DMSO (controls) or IC_50_ and IC_75_ amounts of RK-33. After 72 hours incubation, cells were harvested and stained for Annexin V and PI and analyzed by flow cytometry.

**Figure S3.** **Ki67 expression in CRT tumors**

A, Boxplot displaying percentage of nuclei positive for Ki67 per tumor per treatment group. Median values are displayed with range. Significance was calculated with the Mann-Whitney U-test.

B-E, Example of frequent nuclear Ki67 expression in an untreated tumor at 20X (B) and 40X (C) magnification. Example of infrequent nuclear Ki67 expression in a tumor treated with the combination of radiotherapy and RK-33 at 20X (D) and 40X (E) magnification. Scale bar is 100µm.
