# Supplementary material for: Targeting DDX3 with a small molecule inhibitor for lung cancer therapy
Source: EMBO Mol Med. 2015 Mar 27;7(5):648–69. doi: 10.15252/emmm.201404368 (PMC4492822; doi:10.15252/emmm.201404368)
Supplement: Supplementary file 3 [file emmm0007-0648-sd3.pdf]

EMBO Molecular Medicine

Targeting DDX3 with a small molecule inhibitor for lung cancer therapy

Guus M. Bol, Farhad Vesuna, Min Xie, Jing Zeng, Khaled Aziz, Nishant Gandhi, Anne Levine, Ashley Irving, Dorian Korz, Saritha Tantravedi, Marise R. Heerma van Voss, Kathleen Gabrielson, Evan A. Bordt, Brian M. Polster, Leslie Cope, Petra van der Groep, Atul Kondaskar, Michelle A. Rudek, Ramachandra S. Hosmane, Elsken van der Wall, Paul J. van Diest, Phuoc T. Tran, Venu Raman

Corresponding author: Venu Raman, Johns Hopkins University

Review timeline:

Submission date:	25 June 2014
Editorial Decision:	31 July 2014
Revision received:	03 November 2014
Editorial Decision:	20 November 2014
Revision received:	21November 2014
Editorial Decision:	18 December 2014
Editorial Decision:	27 January 2015
Accepted:	12 February 2015

Transaction Report:

(Note: With the exception of the correction of typographical or spelling errors that could be a source of ambiguity, letters and reports are not edited. The original formatting of letters and referee reports may not be reflected in this compilation.)

Editor: Roberto Buccione

1st Editorial Decision 31 July 2014

Thank you for the submission of your manuscript to EMBO Molecular Medicine. We are sorry that it has taken longer than we would have liked to get back to you on your manuscript. In this case we experienced difficulties in securing three appropriate reviewers and then obtaining their evaluations in a timely manner.

We have now heard back from the three Reviewers whom we asked to evaluate your manuscript. You will see that in aggregate, they raise a number of specific concerns that prevent us from considering publication at this time.

As you will see, while Reviewers 2 and 3 are quite supportive of your study, Reviewer 1 is more critical. I will not discuss each point in detail as they are clearly stated. There are, however, some fundamental points that I feel deserve further clarification on my part.

Reviewer 1 is quite critical and notes that the results presented in this manuscript appear in contrast with a recent paper demonstrating that DDX3 loss promotes malignancy and is associated with poor patient prognosis (PMID: 23584477). I agree that this paper should be cited and discussed in relation to your findings. Reviewer 1 would also like you to confirm the role of DDX3 expression in independent cohorts of patients to consolidate your findings. S/he is also not convinced that IHC

was performed to the highest standards and suggests some remedies to that effect and would like you to extend your screen to different histological variants. The Reviewer would also like you to extend the mechanistic insight but also to consolidate the translational relevance of your work by adding additional models. This Reviewer also lists additional items of concern. Although I consider that extending your analysis of DDX3 expression to additional cohorts of patients and investigating additional mouse models would indeed be valuable additions, I will not be requiring you to perform these experiments (provided all other issues are carefully and fully dealt with). I do, however encourage you to develop your study as far as realistically possible along the lines suggested by Reviewer 1 for your next, revised version to strengthen your findings and increase their impact.

Reviewer 2 is quite positive and raises few, but in my opinion, relevant questions. Among these, s/he would like to know if RK33 also sensitises lung cancers to chemotherapy regimens currently used in the clinic. Reviewer 2 would also like you to show the threshold level of DDX3 expression for responsiveness to RK33 therapy.

Reviewer 3 also mentions a few concerns for your action. S/he would like to see a comparison of DDX3 expression in normal tissues. This Reviewer also mentions a few experimental issues that need to be addressed and/or better explained including the nature of the helicase assay, results of the pull-down assay and whether the toxicity studies reflect actual in vivo active dose levels. In addition to other items, Reviewer 3 also mentions two other important points: s/he would like you to verify whether binding site mutants rescue drug effects and whether and how much activity does RK33 display as a single agent. Clearly, the issues of drug specificity and in vivo efficacy are important and require further work.

Considered all the above, while publication of the paper cannot be considered at this stage, we would be pleased to consider a revised submission, with the understanding that the Reviewers' concerns must be fully addressed as outlined above, with additional experimental data where appropriate and that acceptance of the manuscript will entail a second round of review.

Please note that it is EMBO Molecular Medicine policy to allow a single round of revision only and that, therefore, acceptance or rejection of the manuscript will depend on the completeness of your responses included in the next, final version of the manuscript.

As you know, EMBO Molecular Medicine has a "scooping protection" policy, whereby similar findings that are published by others during review or revision are not a criterion for rejection. However, I do ask you to get in touch with us after three months if you have not completed your revision, to update us on the status. Please also contact us as soon as possible if similar work is published elsewhere.

I look forward to seeing a revised form of your manuscript as soon as possible.

***** Reviewer's comments *****

Referee #1 (Comments on Novelty/Model System):

Recently, Wu et al have published a manuscript demonstrating that DDX3 inactivation promotes tumor malignancy and poor patients' outcome in NSCLC (Oncogene; 2014 33, 1515-1526). The role of DDX3 expression on NSCLC patient prognosis should be determined in independent cohorts in order to substantiate the results presented herein. In my opinion, this is a key experiment that will help to elucidate the precise role of DDX3 in lung cancer.

Referee #1 (Remarks):

The manuscript by Bol et al. follows up their previous observation that DDX3 has a role in breast cancer progression. This group has first evaluated the prognostic role of DDX3 in lung cancer and show that high DDX3 expression is associated with poor survival. Furthermore, they have designed a small molecule inhibitor of DDX3 and evaluate its efficacy in cell lines and mouse models of NSCLC. This is an interesting therapeutic strategy in lung cancer. However, I am particularly

concerned about the divergence between the results presented in this manuscript and those reported recently by Wu et al. These authors claim that high DDX3 expression in NSCLC is associated with better survival and longer recurrence-free survival. This antagonistic question should be addressed before pointing out the inhibition of DDX3 as a therapeutic strategy.

My comments about the study are as follows:
1. Recently, Wu et al have published a manuscript demonstrating that DDX3 inactivation promotes tumor malignancy and poor patients' outcome in NSCLC (Oncogene; 2014 33, 1515-1526). These authors performed soft agar colony studies and invasion assays in A549 cells with DDX3 downregulated by siRNA technology and H1299 cells overexpressing DDX3. They claim that DDX3 knockdown increases soft agar colony size and induces invasion, and the contrary results were found when DDX3 was overexpressed. More importantly, survival analysis of DDX3 expression in NSCLC patients demonstrated opposite results to those observed in the present manuscript. In the multivariate analysis, Wu et al reported that high DDX3 expression was an independent predictor of better survival and longer recurrence-free survival. In addition, Wu et al suggest that DDX3 loss by P53 inactivation promotes tumor malignancy via the MDM2/Slug/E-cadherin pathway. Bibliography should be updated to include this reference.
In order to substantiate their findings, further studies should be done:
1. The role of DDX3 expression on NSCLC patient prognosis should be determined in independent cohorts in order to substantiate the results presented herein. In my opinion, this is a key experiment that will help to elucidate the precise role of DDX3 in lung cancer.
Moreover, specific controls of the immunohistochemical technique need to be performed. Quantification of the staining needs to be done by two independent observers and the method of quantification (only intensity was evaluated?; H score that combines intensity and percentage of positive cells may help to evaluate the results) should be reported in the Methods section. What is the cut-off used to select high and low expression?
Definitely, SCLC and NSCLC are two different entities and survival/recurrence results should be presented separately for both groups.
2. The effect of DDX3 inhibition by RK-33 on proliferation, cell survival and apoptosis (accurately measured by PI/Annexin V measure for example) could be performed in a wide panel of lung cancer cell lines. Moreover, the effect of DDX3 in specific histologies of lung cancer should be analysed. Not only differences between NSCLC and SCLC, but also differences between adenocarcinoma and squamous cell carcinoma.
3. The effect of DDX3 inhibition on downstream signalling should be evaluated in the previously mentioned panel of lung cancer cell lines. Not only ERK activation but also P53 pathway as suggested by Wu et al could be evaluated. Moreover, the increase in ERK phosphorylation in the H3255 cell line (low DDX3 expression) needs to be mentioned and further studied.
4. It is suggested that the authors revise Figure 6 to include additional experimental mouse models to evaluate the effect of RK-33 on tumor growth. Furthermore, specific statistical design should be performed in order to evaluate the synergistic effect of radiotherapy and DDX3 blockade.
5. It would be worthy to extend the study of the Twist1/KrasG12D model. In this sense, proliferation and apoptosis could be detected in tumor cells of control and treated tumor cells.

Other considerations:
1. The statistical methods should be detailed.
2. In the microCt evaluation, and in order to follow RECIST criteria, the longest diameter could also be measured and both volume and diameter measures could be compared. Image size for microCT results is not appropriate; it is difficult to visualize tumors with the actual reduced size of the images.
3. The authors must reorganize this paper so that the narrative follows the figure arrangement more carefully. It would be useful that figures follow some kind of harmony (this impression is more pronounced in figures 1, 2 and 6: figure size could be adjusted in order to give a clearer appearance)
4. Mitochondrial function may be determined by independent methods such as ATP consumption or mitochondrial membrane potential markers.
5. An extensive review of the most recent bibliography should be done (i.e more recent global statistics could be mentioned).
6. Label is missing in figure 5C
7. Page 29, first line: a minor correction should be done here: "Inducible Twist1/KrasG12D transgenic mice [31] were treated as in Fig. 4C and H". I wondered if Figure 6 instead of Fig 4 needs to be cited here.

Referee #2 (Remarks):

EMM-2014-04368
Bol et al. "Targeting DDX3 with a small molecule inhibitor for lung cancer therapy "

The authors study the effect of a small molecule (RK-33) they had previously synthesized that binds to DDX3, on the growth of non-small cell lung cancer (NSCLC0 preclinical models. Genetic silencing of DDX3 is also used. They present data showing DDX3 is over expressed in all histologic types of lung cancer and this is associated with inferior survival. RK-33 in NSCLC cell lines induced G1 arrest, induced apoptosis, and caused significant radio-sensitization in preclinical tumor models in vitro and in vivo and in a genetically engineered mouse model of lung cancer. RK-33 and DDX3 genetic down regulation, inhibited RNA helicase activity, impaired WNT signaling and inhibited non-homologous end joining recombination. RK-33 exhibited no toxicity in their mouse studies.

Comments to the authors:

Overall in these preclinical studies the authors present a compelling case for therapeutic targeting of DDX3 in lung cancers and the use of the small molecule RK-33 as an important starting point for clinical drug development.
1. It would have been interesting to know if RK-33 sensitized lung cancers to any types of chemotherapy or targeted therapy currently used in clinical treatment.
2. It would be of use to know if from deposited datasets DDX2 is amplified or mutated in lung cancer.
3. Presumably high DDX3 expression would be the enrollment biomarker for such targeted therapy. Could the authors summarize their quantitative data on this point from their preclinical models? Is there a threshold level of DDX3 expression required to be responsive to RK-33 targeted therapy?

Referee #3 (Remarks):

The authors report a new inhibitor of DDX3 RNA helicase. This is a good study and it should be published. Some questions for the authors that may help improve the study and I am aware that some suggestions may go beyond the frame of this MS.

1) How was the drug found?
2) Expression data in Fig. 1. There is no comparison to normal tissues, it would perhaps help to include oncomine or cell line encyclopedia expression data, or different tumors. Note that DDX3 has been reported as a mutational target in GBM.
3) A more detailed description of expression in other normal tissues would help anticipate toxicities.
4) Legends for Fig 2 are mislabeled.
5) What is the subsrate in the helicase assay. Have the authors explored RNA secondary (e.g. G-quadruplex or hairpin structures)?
6) The pull down shows DDX3 an the data are very clear. Do other, related proteins come down? E.g. DDX2?
7) If the binding sites are known, can the authors use binding site mutants that would rescue the drug effect. This would
8) Fig. 3 shows gene expression effects. It is likely beyond this MS, but a RNA IP with DDX3 would enhance insight into its activity.
9) Do the toxicity studies reflect an in vivo active dose level?
10) The drug seems to show little single agent activity in a murine model in vivo. Please clarify the single agent activity. Is there efficacy in xenografts of lines treated in vitro?
11) Fig. 7. Please add a quantification of cell death.
12) Effects on Wnt are interesting but seem somewhat disconnected from the MS. (or I missed the link).

Overall, an interesting study and a potentially interesting new compound. The study would be even more impactful if the authors could show resistance in binding site mutants to demonstrate that the effect actually relates to DDX3 and if they could demonstrate single agent activity at a non-toxic dose level in vivo.

1st Revision - authors' response 03 November 2014

We thank the reviewers for their comments and as per their suggestions, we have added significant new data to address the queries raised. The point-by-point responses to the individual queries are indicated below.

Reviewer 1

1) Moreover, specific controls of the immunohistochemical technique need to be performed.
Response: A detailed description of the controls used and the techniques employed are now included in the Methods Section titled "**Immunohistochemistry**".

2) Quantification of the staining needs to be done by two independent observers and the method of quantification.
Response: The immunostained slides have now been scored by two independent pathologists. This is indicated in the Methods Section titled " **Scoring of immunohistochemistry**". The inter-rater reliability (Cohen's Kappa) between both pathologists is 0.743, which is mentioned in the Method Section titled "**Clinical samples**".

3) H score that combines intensity and percentage of positive cells may help to evaluate the results) should be reported in the Methods section.
Response: We have now determined the H score, which was similar to cytoplasmic intensity of DDX3. Cytoplasmic DDX3 is homogeneously distributed throughout the sample, as demonstrated in Figure 1F. The calculated H-scores, as expected, did not significantly differ from intensity of cytoplasmic DDX3 staining. Because intensity scores would be clinically be to interpret outcome, we have used this for the results, presented in this paper. (This is also added to the Methods Section titled " **Scoring of immunohistochemistry**".

4) What is the cut-off used to select high and low expression?
Response: We agree with the reviewer that knowing such a threshold is of great value to most effectively select those patients likely to benefit from RK-33 treatment. We have clear *in vitro* evidence that cell lines with high DDX3 expression (H460 and A549) are more responsive to RK-33 than a cell line with low DDX3 expression (H3255). The preclinical mouse models we used to determine efficacy were all models with high DDX3 expressing tumors, which were sensitive to RK-33 treatment. We have also found that DDX3 expression levels are objectively assessable using immunohistochemistry in a clinical setting. This allows for evaluation of efficacy in groups of patients with varying DDX3 expression levels in early clinical trials, after which we anticipate to be able to establish a definitive cut-off for RK-33 sensitivity. DDX3 scores 1 and 2 were grouped as low DDX3 expression and evaluated against high DDX3 expression (score = 3). Cytoplasmic expression of DDX3 was uniform within a single tumor (Figure 1) as shown by similar H-scores and intensity scores of DDX3 expression. Only intensity scores are shown. This is indicated in the Methods Section titled "**Scoring of Immunohistochemistry**".

5) Definitely, SCLC and NSCLC are two different entities and survival/recurrence results should be presented separately for both groups.
Response: As can be seen in Figure 1J, the majority of the samples we analyzed were NSCLC (SCLC = 6, NSCLC = 86). The corresponding survival curve is shown in Figure 1K. Given the small sample set of SCLC, we were unable to draw a survival curve that will project any meaningful information.

6) The effect of DDX3 inhibition by RK-33 on proliferation, cell survival and apoptosis (accurately measured by PI/Annexin V measure for example) could be performed in a wide panel of lung cancer cell lines.
Response: We have now carried out these experiments as requested by the reviewer in multiple lung cancer cell lines (H23, H358, H1299, A549, H196, and H460). This information is included in the Expanded View, Figure E1.

7) Not only differences between NSCLC and SCLC, but also differences between adenocarcinoma and squamous cell carcinoma.
Response: From a clinical perspective these are indeed essential questions to ask before RK-33 will be used in cancer care. However, in our opinion, that is beyond the scope of this manuscript. This study was never intended to do extensive subgroup analysis; hence this study population lacks the power to do so. However, in Table 1 A and B we do show DDX3 expression within the different histological subclasses of lung cancer. Also, please see the response to query 5.

8) The effect of DDX3 inhibition on downstream signaling should be evaluated in the previously mentioned panel of lung cancer cell lines. Not only ERK activation but also p53 pathway as suggested by Wu et al could be evaluated.
Response: As requested by the reviewer, we did evaluate the role of p53 in the DDX3 pathway. As shown below, even in the isogenic cell lines HCT116$^{p53+/+}$ and HCT116$^{p53-/-}$, devoid of the p53 gene, there is no perturbation of DDX3 expression. As loss of p53 did not result in loss of DDX3 expression, the association with MDM2/Slug/E-cadherin pathway is unclear within our experimental setting. In support of our finding, we have earlier published the lack of transcriptional activation of p21 by DDX3 (Botlagunta et al, 2008). Also, there is a report (Sun et al, 2011), which indicates that DDX3 expression is required for Snail expression and may drive tumorigenesis in some cancers.

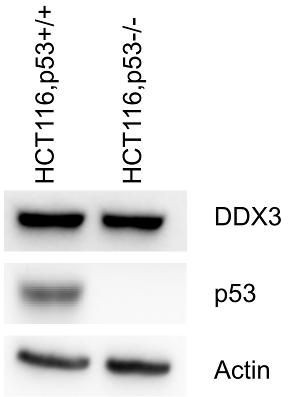

Total proteins were extracted from the cell lines and scored for DDX3, p53 and actin.

9) Moreover, the increase in ERK phosphorylation in the H3255 cell line (low DDX3 expression) needs to be mentioned and further studied.
Response: We have indicated the increase in ERK phosphorylation in H3255 cell line in the Results Section titled "**RK-33 induces G1 arrest and causes apoptosis".** Further characterization of ERK activity in conjunction with DDX3 expression and RK-33 will be part of a different study.

10) Furthermore, specific statistical design should be performed in order to evaluate the synergistic effect of radiotherapy and DDX3 blockade.
Response: We have now clarified this in the text. The *in vitro* calculations were done using the method developed by Chou and Talalay (Chou, 2010). For the *in vivo* work, we have re-worded to indicate that we have seen greater than additive effect in the combination treatment as compared to the individual treatment regimen. Also, to the best of our knowledge there are no statistical methods to do this on *in vivo* datasets.

11) It would be worthy to extend the study of the Twist1/KrasG12D model. In this sense, proliferation and apoptosis could be detected in tumor cells of control and treated tumor cells.

Response: As suggested by the reviewer, we carried out immunohistochemistry analysis for caspase 3 and Ki67 on the lung tumors from the Twist1/KrasG12D model following treatment (please see Expanded View Figure E2). Even though we saw a decrease in Ki67 positive cells following RK-33 and radiation treatment, it would be difficult to discern the exact nature of the effect as we are not analyzing the tumor samples immediately after treatment. It is possible that tumor normalization has occurred, thus decreasing the observable effect, in this case, Ki67 expression. Moreover, the percentage of cells stained for caspase 3 was below 1% of the cell population, thus making a meaningful interpretation difficult.

12) The statistical methods should be detailed.
Response: We have now indicated the statistical methods in the Methods Section titled "**Statistics**".

13) In the microCt evaluation, and in order to follow RECIST criteria, the longest diameter could also be measured and both volume and diameter measures could be compared. Image size for microCT results is not appropriate; it is difficult to visualize tumors with the actual reduced size of the images.
Response: The co-author, Phuoc Tran, MD, PhD, whose laboratory performed this analysis, is a board-certified radiation oncologist who is also a PI on clinical protocols utilizing RECIST criteria. The RECIST is a convenient human tumor response tool that is used in clinical trials as a compromise between sensitivity for a signal and ease of use across many different institutions and radiologists of varying levels of experience. It was never meant to be a final answer to image analysis in the clinic and certainly not in the pre-clinical domain. The tumor quantification performed has been previously published by the co-author (Tran et al, 2011; Zeng et al, 2013) and utilizes pre-clinical tumor volumes calculated from genetically engineered mouse models directly by contouring the lesion slice by slice with confirmation using complimentary orthogonal views from a imaging analysis workstation. We have clarified this by including a paragraph on image analysis in the revised version in the Methods Section titled "**Micro-CT image analysis**".

14) The authors must reorganize this paper so that the narrative follows the figure arrangement more carefully. It would be useful that figures follow some kind of harmony (this impression is more pronounced in figures 1, 2 and 6: figure size could be adjusted in order to give a clearer appearance.
Response: We appreciate the suggestions by the reviewer. We have implemented these changes in the text.

15) Mitochondrial function may be determined by independent methods such as ATP consumption or mitochondrial membrane potential markers.
Response: As suggested by the reviewer, we have carried out experiments to demonstrate that a therapeutically relevant RK-33 concentration does not interfere with mitochondrial function. We evaluated ATP levels in HAPI cells incubated with the cell permeable mitochondrial complex I substrate pyruvate (10mM) in the absence of glucose and the presence of the glycolysis inhibitor 2-deoxyglucose (50mM) [Figure 5F]. Under these conditions, the majority of cellular ATP was generated by mitochondria, as demonstrated by addition of the ATP synthase inhibitor oligomycin which significantly decreased total cellular ATP by ~70%. In contrast to oligomycin, RK-33 (10μM) failed to significantly decrease ATP. Thus, RK-33 has no effect on either mitochondrial respiration or ATP generation.

16) An extensive review of the most recent bibliography should be done (i.e. more recent global statistics could be mentioned).
Response: We have addressed this in the revised manuscript by including the latest global statistics.

Reviewer 2

1) It would have been interesting to know if RK-33 sensitized lung cancers to any types of chemotherapy or targeted therapy currently used in clinical treatment.
Response: We did combination experiments with RK-33 + cisplatin and RK-33 + paclitaxel on multiple lung cancer cell lines (A549, H1299, H460, H23, and H196). From the data obtained, we did not observe significant synergy with either of these two drugs. This is in parallel to data obtained when aligning RK-33 with other FDA approved oncological drugs (Figure 4C-D).

2) It would be of use to know if from deposited datasets DDX2 is amplified or mutated in lung cancer.
Response: According to the COSMIC database, the mutation rates of DDX3 range from 2-3%. Amplification rates are below 1%.

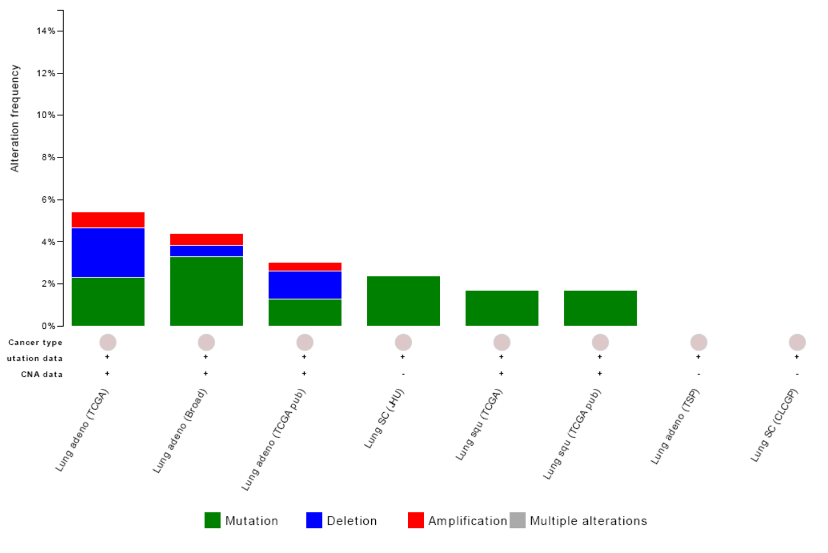

DDX3 mutation rates from various datasets as seen in the COSMIC database.

Adenocarcinoma
Broad (Imielinski et al, 2012)
Somatic mutation rate: 3.3%

TCGA, Nature, in press
Somatic mutation rate: 1.3%

TCGA, Provisional
Somatic mutation rate: 2.3%

TSP (Ding et al, 2008)
Somatic mutation rate: 0%

Squamous carcinoma
TCGA, Nature (CGAR, 2012)
Somatic Mutation Rate: 1.7%

TCGA, Provisional
Somatic Mutation Rate: 1.7%

3) Presumably high DDX3 expression would be the enrollment biomarker for such targeted therapy. Could the authors summarize their quantitative data on this point from their preclinical models? Is there a threshold level of DDX3 expression required to be responsive to RK-33 targeted therapy?
Response: Please see response to query 4 of Reviewer 1.

Reviewer 3

1) How was the drug found?
Response: RK-33 was rationally designed. Please see Results and Figure 2A-C, in the manuscript.

2) Expression data in Figure 1. There is no comparison to normal tissues, it would perhaps help to include oncomine or cell line encyclopedia expression data, or different tumors.
Response: Figure 1F displays normal lung tissue, which we used for comparison with lung tumors. Also, in Figure 1A we have shown that the normal lung epithelial cell line, HBEC, has little or no DDX3 protein expression. Searching the Oncomine database, we found that DDX3 expression

levels vary based on the datasets analyzed. In addition, as far as DDX3 protein levels are concerned, it should be noted that there is no 1:1 correlation between the DDX3 mRNA and protein levels, thus making it difficult to interpret the gene expression datasets as an absolute unit to that of the protein levels.

3) A more detailed description of expression in other normal tissues would help anticipate toxicities.
Response: It is difficult to ascertain if the levels of DDX3 in the normal cells will directly correlate to toxicities levels following RK-33 treatment. It is possible that normal cells are not dependent on DDX3 for its cellular biogenesis as compared to the cancer cells. This could be one of the reasons why there is no observable toxicity within our experimental setting. Also, as a first step to determine toxicity for a novel drug, it is essential to test the effect in small animals like mouse or rat. Based on these criteria, we observed no toxicity in mouse at higher levels than the therapeutic dose to kill cancer cells. This indicates that perturbation of DDX3 levels by RK-33 in normal cells does not impart any visible side effects.

4) Legends for Figure 2 are mislabeled.
Response: We thank the reviewer for pointing this out. We have rectified this in the revised manuscript.

5) What is the subsrate in the helicase assay. Have the authors explored RNA secondary (e.g. G-quadruplex or hairpin structures)?
Response: The substrate used is now included in the Methods Section titled "**Helicase assay**". With respect to exploring RNA secondary structures as possible substrates, we have yet to determine the optimal sequence. Initial attempts did not exhibit any robust unwinding of hairpin structures in our *in vivo* reporter assays. Experiments are ongoing to identify specific secondary structures that will be unwound by DDX3.

6) The pull down shows DDX3 and the data are very clear. Do other, related proteins come down? E.g. DDX2?
Response: As suggested by the reviewer, we scored for two other DDX3 related proteins, DDX5 and DDX17, following pull-down. As shown in the revised Figure 2F, it appears from the panel of proteins we tested, RK-33 is specific for DDX3.

7) If the binding sites are known, can the authors use binding site mutants that would rescue the drug effect.
Response: Even though the putative binding site of RK-33 to DDX3 can be determined, the kinetic of binding studies is beyond the scope of this manuscript and will form part of another study. However with respect to the specificity of RK-33, we have now included data, which indicates that RK-33 preferentially binds to DDX3 when compared to other RNA helicase family members such as DDX5 and DDX17. Also, we have data to indicate that lung cancer cell line, H3255, which has very little DDX3 expression is not responsive to RK-33 at the dose tested. Importantly, RK-33 did not exhibit any toxicity in mice. Taken together this indicates that RK-33 demonstrates a great deal of specificity for DDX3, validating our approach of the use of rational drug design.

8) Do the toxicity studies reflect an *in vivo* active dose level?
Response: It does reflect *in vivo* active dose. 20 mg/Kg of RK-33 results in therapeutic dose in various tissues that we tested (Figure 5A-B).

9) The drug seems to show little single agent activity in a murine model *in vivo*. Please clarify the single agent activity. Is there efficacy in xenografts of lines treated *in vitro*?
Response: As our goals for this manuscript were to expand the radiosensitizing ability of RK-33, we did have not carry out single agent efficacy in our preclinical lung cancer models. However, we did observe single agent activity in our preclinical breast and sarcoma models, which resulted in delayed tumor growth.

10) Figure 7. Please add a quantification of cell death.
Response: As suggested by the reviewer, we tested the effect of RK-33 on six lung cell lines and assayed for cell death by expression of Annexin V and PI using flow cytometry. The results are displayed in Figure E1.

References:

Botlagunta M, Vesuna F, Mironchik Y, Raman A, Lisok A, Winnard P, Jr., Mukadam S, Van Diest P, Chen JH, Farabaugh P et al (2008) Oncogenic role of DDX3 in breast cancer biogenesis. Oncogene 27**:** 3912-3922

Chou TC (2010) Drug combination studies and their synergy quantification using the Chou-Talalay method. Cancer Res 70**:** 440-446

Ding L, Getz G, Wheeler DA, Mardis ER, McLellan MD, Cibulskis K, Sougnez C, Greulich H, Muzny DM, Morgan MB et al (2008) Somatic mutations affect key pathways in lung adenocarcinoma. Nature 455**:** 1069-1075

Imielinski M, Berger AH, Hammerman PS, Hernandez B, Pugh TJ, Hodis E, Cho J, Suh J, Capelletti M, Sivachenko A et al (2012) Mapping the hallmarks of lung adenocarcinoma with massively parallel sequencing. Cell 150**:** 1107-1120

Network CGAR (2012) Comprehensive genomic characterization of squamous cell lung cancers. Nature 489**:** 519-525

Sun M, Song L, Zhou T, Gillespie GY, Jope RS (2011) The role of DDX3 in regulating Snail. Biochim Biophys Acta 1813**:** 438-447

Tran PT, Bendapudi PK, Lin HJ, Choi P, Koh S, Chen J, Horng G, Hughes NP, Schwartz LH, Miller VA et al (2011) Survival and death signals can predict tumor response to therapy after oncogene inactivation. Science translational medicine 3**:** 103ra199

Zeng J, Aziz K, Chettiar ST, Aftab BT, Armour M, Gajula R, Gandhi N, Salih T, Herman JM, Wong J et al (2013) Hedgehog pathway inhibition radiosensitizes non-small cell lung cancers. Int J Radiat Oncol Biol Phys 86**:** 143-149

2nd Editorial Decision 20 November 2014

Thank you for the submission of your revised manuscript to EMBO Molecular Medicine.

Since Reviewer 2 was unavailable, I asked Reviewers 1 and 3 to also evaluate your responses to the former. We have now received the enclosed reports from the two Reviewers that were asked to re-assess your manuscript.

As you will see, while Reviewer 3 is now globally supportive, Reviewer 1 still has a few remaining issues.

Briefly, Reviewer 1 would like you to substantiate your claim of the prognostic value of DDX3 expression by validating it on a independent cohort of patients. The Reviewer also admits that it might be difficult to do so with patient samples but suggests the use of published mRNA expression databases for NSCLC. I feel that the Reviewer has a valid point and I also note that you had not omitted to deal with this specific request in your rebuttal. On the other hand, I recognise that this is not the core finding of your work. In conclusion, I must ask you to provide a direct response to the Reviewer. In addition, and ideally, I would encourage you to provide additional validation data to support your claim of the prognostic values of DDX3 expression, as indicated by Reviewer 1; In alternative, I would suggest you to tone down your claim as appropriate. Please note that Reviewer 1 also reiterates his/her request to provide larger microCT images.

Please also consider the following final Editorial amendments/requests for your revision:

1) EMBO Molecular Medicine now requires a complete author checklist (http://embomolmed.embopress.org/authorguide#editorial3) to be submitted with all revised

manuscripts. Provision of the author checklist is mandatory at revision stage; The checklist is designed to enhance and standardize reporting of key information in research papers and to support reanalysis and repetition of experiments by the community. The list covers key information for figure panels and captions and focuses on statistics, the reporting of reagents, animal models and human subject-derived data, as well as guidance to optimise data accessibility. I am attaching a copy of the checklist to this letter for your convenience, but should you have difficulties opening it, please refer to the link above.

2) Every published paper now includes a 'Synopsis' to further enhance discoverability. Synopses are displayed on the journal webpage and are freely accessible to all readers. They include a short standfirst - to be written by the editor - as well as 2-5 one-sentence bullet points that summarise the paper (to be written by the author). Please provide the short list of bullet points that summarise the key NEW findings. The bullet points should be designed to be complementary to the abstract - i.e. not repeat the same text. We encourage inclusion of key acronyms and quantitative information. Please use the passive voice. Please attach these in a separate file or send them by email, we will incorporate them accordingly

3) We are now encouraging the publication of source data, particularly for electrophoretic gels and blots, with the aim of making primary data more accessible and transparent to the reader. Would you be willing to provide a PDF file per figure that contains the original, un-cropped and unprocessed scans of all or at least the key gels used in the manuscript? The PDF files should be labelled with the appropriate figure/panel number, and should have molecular weight markers; further annotation may be useful but is not essential. The PDF files will be published online with the article as supplementary "Source Data" files. If you have any questions regarding this just contact me.

Please submit your revised manuscript as soon as possible.

I look forward to seeing your revised manuscript.

***** Reviewer's comments *****

Referee #1 (Comments on Novelty/Model System):

Referee #1:
In the revised submission, Bol et al have adequately addressed mainly all the concerns raised in my initial review. However, authors have not solved the question of how reproducible are their results on the prognostic role of DDX3 expression. The use of an independent cohort is essential to substantiate their results. Although I understand that this may be difficult to obtain such independent series of patients, authors may use already published databases of mRNA expression in NSCLC patients that also provide survival data. I would like to emphasize my suggestion of enlarging the size of microCT images.

Referee #2:
I was asked by the Editor to evaluate the Authors' response to Reviewer 2's concerns. In my opinion, the Authors have satisfactorily addressed them.

Referee #3 (Remarks):

The authors have addressed all concerns. Nice study.

2nd Revision - authors' response 21 November 2014

As requested by reviewer 1, we did carry out survival analysis on NSCLC patients with DDX3 expression. We have now included this as an expanded figure in the manuscript. The data supports our analysis, which indicates that high expression of DDX3 in NSCLCs correlates to shorter survival. In addition, we have enlarged the microCT images by 25% as requested by the reviewer.

We hope that the current revised manuscript will be acceptable for publication in EMBO MOLECULAR MEDICINE.

3rd Editorial Decision 18 December 2014

Technical and editorial revision.

4th Editorial Decision 27 January 2015

Thank you for the submission of your revised manuscript to EMBO Molecular Medicine. We will be able to accept your manuscript pending the following final amendments and requests:

1) Please submit 1 (ONE) source data file (with sufficient annotations to allow identification) per manuscript figure.

2) As per our Author Guidelines, the description of all reported data that includes statistical testing must state the name of the statistical test used to generate error bars and P values, the number (n) of independent experiments underlying each data point (not replicate measures of one sample), and the actual P value for each test (not merely 'significant' or 'P < 0.05').

3) The manuscript must include a statement in the Materials and Methods identifying the institutional and/or licensing committee approving the experiments, including any relevant details (like how many animals were used, of which gender, at what age, which strains, if genetically modified, on which background, housing details, etc). We encourage authors to follow the ARRIVE guidelines for reporting studies involving animals. Please see the EQUATOR website for details: http://www.equator-network.org/reporting-guidelines/improving-bioscience-research-reporting-the-arrive-guidelines-for-reporting-animal-research/. I note that you have confirmed compliance in the checklist but please report the above details directly in the manuscript.

4) Please provide a running title and 5 keywords

Please submit your revised manuscript within two weeks. I look forward to seeing a revised, final form of your manuscript as soon as possible.

© EMBO
